# Self-Organization Emerging from Marangoni and Elastocapillary Effects Directed by Amphiphile Filament Connections

**DOI:** 10.1021/acs.langmuir.2c01241

**Published:** 2022-08-25

**Authors:** Mitch Winkens, Peter A. Korevaar

**Affiliations:** Institute for Molecules and Materials, Radboud University, Heyendaalseweg 135, 6525 AJ Nijmegen, The Netherlands

## Abstract

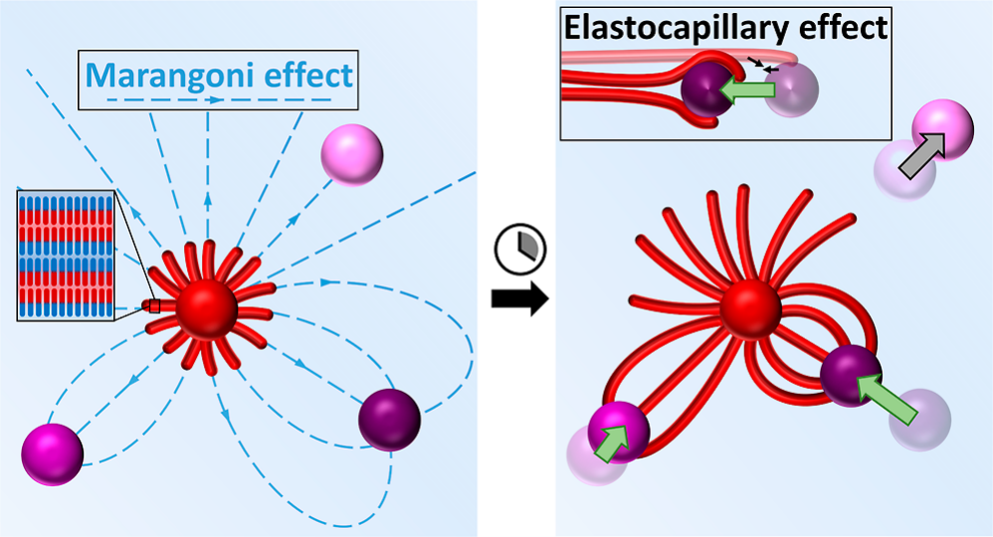

Self-organization of meso- and macroscale structures is a highly active research field that exploits a wide variety of physicochemical phenomena, including surface tension, Marangoni flow, and (elasto)capillary effects. The release of surface-active compounds generates Marangoni flows that cause repulsion, whereas capillary forces attract floating particles via the Cheerios effect. Typically, the interactions resulting from these effects are nonselective because the gradients involved are uniform. In this work, we unravel the mechanisms involved in the self-organization of amphiphile filaments that connect and attract droplets floating at the air–water interface, and we demonstrate their potential for directional gradient formation and thereby selective interaction. We simulate Marangoni flow patterns resulting from the release and depletion of amphiphile molecules by source and drain droplets, respectively, and we predict that these flow patterns direct the growth of filaments from the source droplets toward specific drain droplets, based on their amphiphile depletion rate. The interaction between such droplets is then investigated experimentally by charting the flow patterns in their surroundings, while the role of filaments in source–drain attraction is studied using microscopy. Based on these observations, we attribute attraction of drain droplets and even solid objects toward the source to elastocapillary effects. Finally, the insights from our simulations and experiments are combined to construct a droplet-based system in which the composition of drain droplets regulates their ability to attract filaments and as a consequence be attracted toward the source. Thereby, we provide a novel method through which directional attraction can be established in synthetic self-organizing systems and advance our understanding of how complexity arises from simple building blocks.

## 1 Introduction

Self-organization is pivotal to the formation of functional structures in life—varying from the cytoskeleton that controls cell division^1,2^ to slime mold networks that optimize their nutrient acquisition^3^ and interconnected neurons that establish the brain function.^4^ Synthetic systems can help us to understand how such complex phenomena can emerge from the interactions between a minimalistic set of building blocks.^5^ At the same time, the capability to program self-organization provides paradigms to create novel types of matter that spontaneously generate functional patterns and structures.^6−9^ Pattern generation from initially homogeneous solutions has been established in reaction–diffusion systems such as the Belousov–Zhabotinsky reaction^10,11^ and the thio–urea reaction,^12^ as well as via Rayleigh-Bénard convection that employs minute substrate–product buoyancy differences in an enzymatic reaction network.^13^ Self-organization of complex microstructures has been demonstrated in inorganic precipitation reactions, directed by gradients that emerge around the growing structures.^14,15^ Furthermore, the self-organization of complex flow patterns has been established from active matter, for example, in vitro solutions of microtubules that are moved by cytoskeletal motors^16^ or colloidal dispersions that display diffusiophoretic motility in self-generated ion gradients.^17^

Collective behavior that emerges from Marangoni flow-based systems offers an attractive pathway to establish self-organization of elements that move individually. The Marangoni effect drives a liquid to flow from regions with low surface tension to those with high surface tension.^18−20^ Upon release of a surface-active compound, floating objects can initiate Marangoni flows in the surrounding fluid, such that the objects repel each other and self-organize into a pattern. In the case of a liquid droplet immersed in water, a surfactant that is absorbed or generated at the droplet interface generates a difference in interfacial tension between the front and rear of the droplet. This imbalance results in an internal Marangoni flow, which propels the droplet toward the side with the highest surfactant concentration.^18,19,21^ Collectively, such droplets self-organize into swarms that form dynamic structures,^22−24^ display motion resembling predatory behavior,^25,26^ transfer information,^27^ or solve mazes.^28,29^

Alternatively, self-organizing elements can interact via capillary effects, which involve the deformation of a liquid interface caused by objects coming into contact with it.^30^ Capillary forces have been demonstrated to deform elastic microstructures,^31,32^ even causing them to collapse into hierarchical assemblies.^33,34^ Furthermore, floating objects with lower density than the supporting liquid will be surrounded by either an upward or downward meniscus, if wetting is favorable or unfavorable, respectively,^35,36^ whereas floating objects of higher density are always surrounded by a downward meniscus.^37^ To minimize the air–water interfacial area, floating objects with menisci of equal sign attract one another, a phenomenon known as the “Cheerios effect” which enables the self-organization of floating objects.^23,38,39^

Typically, self-organization relies on the emergence of gradients, for example, in concentration (reaction–diffusion and phoretic effects), surface tension (Marangoni effect), or surface curvature (capillary effect).^35,40,41^ A common feature of these gradients is their uniform, nondirectional spreading; mechanisms by which self-organization is mediated through specific, directional connections between the elements—where some elements attract each other while others are repelled—are seldom reported.

**Figure 1:**
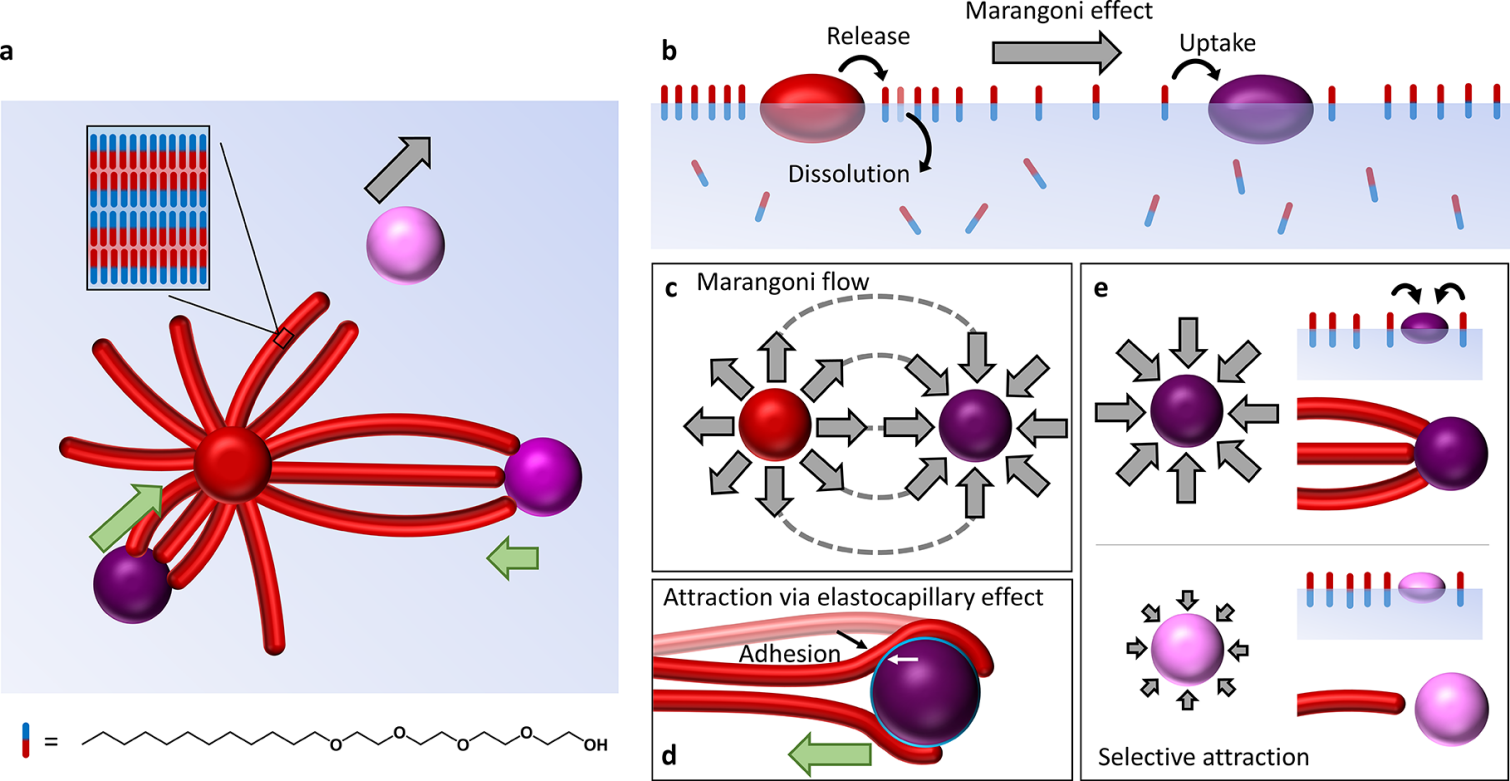
Self-organization mediated by a balance of repulsive and attractive forces. (a) C_12_E_4_ filaments (red rods) grow from the source droplet (red sphere) toward selected drain droplets (purple spheres; darker shades indicate higher amphiphile uptake rate). (b) C_12_E_4_ (red-blue rods) is released from the source to the air–water interface and subsequently dissolved into the aqueous layer or taken up by the drain (side view). The resulting gradient in amphiphile concentration generates an interfacial flow from the source to drain due to the Marangoni effect, which repels the drain from the source. (c) Scheme of Marangoni streamlines leading from the source to drain (top view). (d) Elastocapillary effects cause filaments to wrap around a droplet. If the adhesive forces involved are strong enough, they attract the drain toward the source (top view). (e) Tuning the amphiphile depletion rate at the drains affects the strength of the Marangoni flow, resulting in drains that are selectively attracted by the filaments (top panel) or not attracted (bottom).

In this paper, we investigate how Marangoni flow and capillary effects together establish self-organization at air–water interfaces, via self-assembled filaments that introduce directionality in these interactions. Recently, our research group published a droplet-based system with the ability to self-organize into networks connected by millimeter-long filaments.^42^ These filaments grow from a source droplet of the amphiphile tetra(ethylene glycol) monododecyl ether (C_12_E_4_) that is deposited at an air–water interface. The amphiphile self-assembles into a lamellar phase composed of closely packed bilayers at the boundary of the droplet; the spaces in between these bilayers take up water, and the resulting osmotic pressure forces the bilayers to buckle and form multilamellar filaments—known as “myelin figures” in the literature—which progress over the air–water interface (Figure 1a).^43,44^ Furthermore, the source droplet releases free C_12_E_4_ molecules to the air–water interface. This interfacial C_12_E_4_ is slowly depleted due to its desorption into the underlying aqueous phase, which drives a Marangoni flow that helps to extrude the growing filaments from the source.^45^ When a floating drain droplet of a hydrophobic liquid is deposited, it depletes C_12_E_4_ from the air–water interface as well. The resulting surface tension gradient generates a Marangoni flow that directs the filaments toward the drain (Figure 1b). Upon arrival of the filaments, the drain is observed to be drawn toward the source.

We anticipate that our system has the potential to establish selective connections in self-organization. These connections attract particular floating droplets by distinguishing their chemical composition, which impacts the surrounding surface tension gradient, whereas other droplets are pushed away. We reasoned that the Marangoni flow can be made to favor a particular drain, directing filaments to connect it with the source. First, we develop a model to relate the amphiphile release and depletion dynamics at the air–water interface to the Marangoni flow patterns among source and drain droplets, based on the kinetic rate constants involved. These flow patterns, which direct the filaments from the source to drain, are studied experimentally as well (Figure 1c). Next, we investigate the elastocapillary forces by which the filaments attract the drain droplets toward the source upon connection (Figure 1d). Guided by the model, we assess how the amphiphile depletion rate at the drain droplet, depending on its chemical composition, determines the flow pattern that is followed by the filaments (Figure 1e). Finally, these insights allow us to construct a system in which filaments selectively connect the source to particular drain droplets and thereby demonstrate self-organization via directional interactions.

## 2 Experimental Section

### Materials

Tetra(ethylene glycol) monododecyl ether was purchased from United States Pharmacopeia (100.0%) and Santa Cruz Biotechnology Inc. (Dallas, TX) (≥98.0%). Sodium alginate, Oil red O, 4 Å molecular sieves (4–8 mesh), Solvent green 3 (≥95%), red fluorescent sulfate-modified polystyrene beads (0.1 μm, aqueous suspension), and red fluorescent carboxylate-modified polystyrene beads (0.5 μm, aqueous suspension) were purchased from Sigma-Aldrich, sodium chloride (≥99.6%) was purchased from Fisher Chemical, oleic acid was purchased from Fluorochem (≥95%) and Sigma-Aldrich (≥99%), and sodium oleate (≥95%) was purchased from ABCR GmbH (Karlsruhe, Germany). All materials were used as received unless otherwise noted.

### Filament-Mediated Self-Organization

Filament growth was initiated following a procedure reported previously,^42^ by depositing a 1.0 μL C_12_E_4_ droplet with a Gilson pipette at the interface of the medium solution in a polystyrene Petri dish (lid of a Falcon 35 mm dish, diameter 38 mm, height 4.5 mm, used as received). In order to keep the droplet from moving toward the solution meniscus at the edge of the Petri dish, the dish was generally filled completely with 5.5 mL of medium solution, consisting of sodium alginate (NaAlg, 6.25 mg/mL) and sodium chloride (NaCl, 17 mM) in MilliQ water, such that a convex air–water interface was formed. NaAlg is included to increase the viscosity, and NaCl is included to enhance the stability of the sodium oleate/oleic acid drain droplets.^42^ For the experiment using molecular sieves, 4.0 mL of medium solution was put into a polystyrene Petri dish (Falcon 35 mm, height 9.5 mm, used as received) instead to create a concave air–water interface. Prior to the deposition of any source droplet, the surface tension of the medium solution was decreased by touching the interface with a C_12_E_4_-loaded pipette tip. All experiments were performed at room temperature. The formation of the lamellar phase of C_12_E_4_ in our experiments was verified by the opaque appearance, to the naked eye, of the source droplet after deposition on the medium solution. 1.0 μL drain droplets or 4 Å molecular sieves were deposited shortly after growth of filaments had started. Further details regarding the microscopy experiments are described in the Supporting Information.

### Surface Tension Measurements

The Wilhelmy plate technique was used to measure the interfacial tension at the air/water interface in the center of a polystyrene Petri dish (Falcon 35 mm, height 9.5 mm, used as received) containing 5 mL of NaCl (17 mM) and C_12_E_4_ (522 μM) in water. The setup was left to equilibrate for a minimum of 12 min, after which 1.0 μL of drain solution (or a 4 Å molecular sieve) was deposited near the wall of the Petri dish. Except for the 4 Å molecular sieve, which remained afloat in between the platinum plate and the wall, all drains moved toward the meniscus at the edge of the Petri dish.

### Particle Image Velocimetry Experiments

For top-view particle image velocimetry (PIV) analysis of the flow in the medium, 25 ppm (w/w) red fluorescent carboxylate-modified polystyrene beads were dispersed in the medium. For side-view imaging of the medium, 50 ppm (w/w) red fluorescent sulfonate-modified polystyrene beads were included instead. For PIV analysis of the flow inside of the drain, an aqueous suspension of red fluorescent carboxylate-modified polystyrene beads was freeze-dried prior to inclusion inside the drain at 250 ppm (w/w) concentration. PIV analysis was performed on the uncompressed versions of Movies S1–S3, S7, and S10–S13, which can be provided upon request, using version 2.38 of the *PIVlab* plugin for Matlab (R2019b).^46,47^ Further details regarding the methodology are described in the Supporting Information.

### Tracking of Floating Objects

In Figure 5d, the trajectories of the source droplet, molecular sieve, and filament defects were traced using the Trackmate plugin for Fiji.^48,49^ Further details regarding the methodology are described in the Supporting Information.

### Simulation of Marangoni Flow Patterns

The surface tension dynamics and the Marangoni flow patterns are simulated using Matlab (R2017a). A full description of the model and parameters used in the simulations is provided in the Supporting Information.

## 3 Results and Discussion

### 3.1 Simulating the Flow Patterns Based on Amphiphile Release and Depletion Dynamics

**Figure 2:**
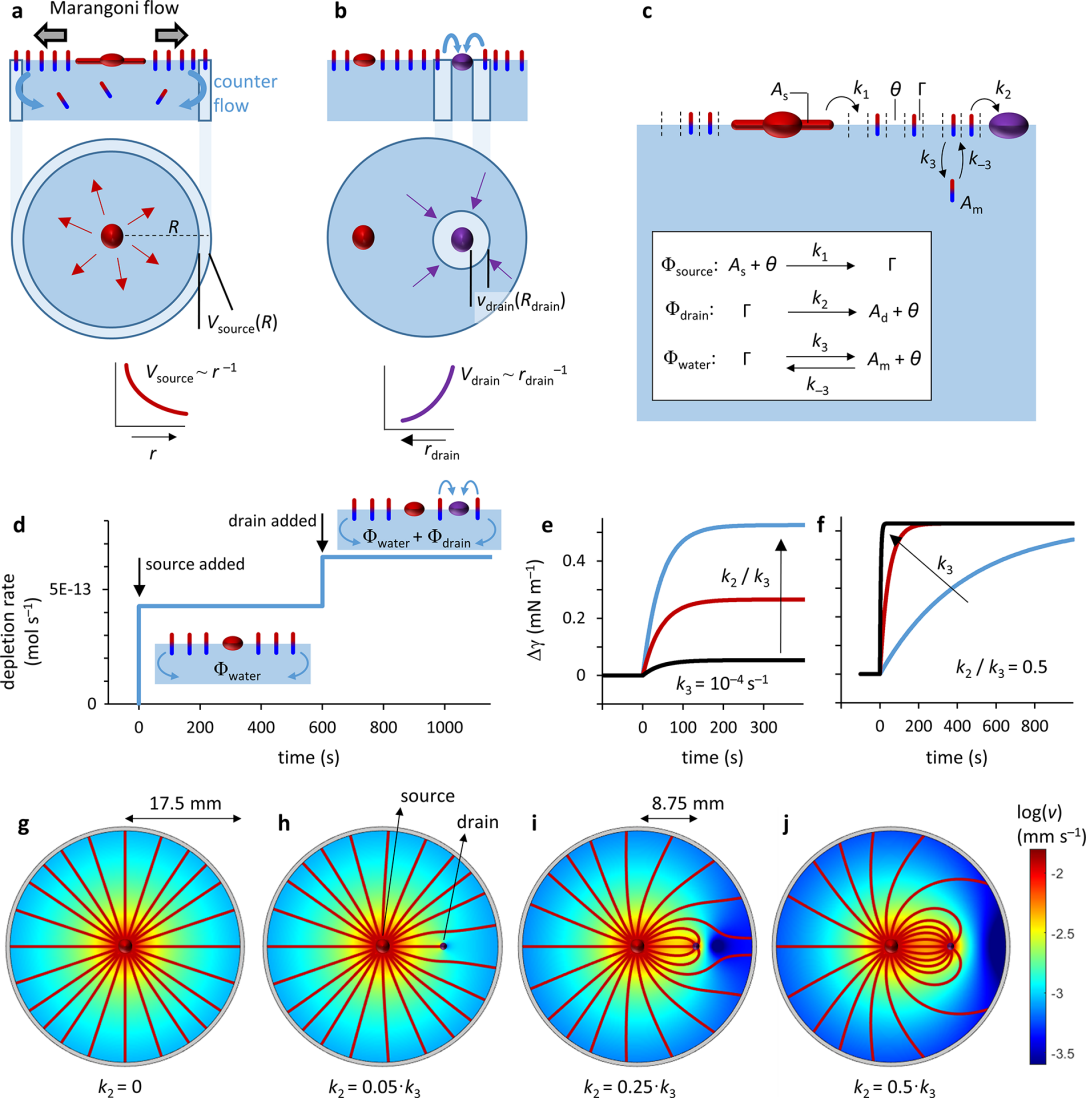
Simulations of the Marangoni flow patterns between source and drain droplets. (a,b) Depletion of amphiphile from the air–water interface occurs via the Marangoni convection in a ring at the boundary of the flow zone (a) and around the drain droplet (b). (c) Kinetic model describing the depletion of amphiphiles from the air–water interface toward the aqueous phase (Φ_water_) and the drain (Φ_drain_). (d) Simulation of the amphiphile depletion rate upon deposition of a source droplet at *t* = 0 s and a drain droplet at *t* = 600 s. (e,f) Simulations of the surface tension Δγ for a C_12_E_4_ solution (0.52 mM) upon deposition of a drain droplet at *t* = 0 s, for (e) *k*_2_/*k*_3_ = 0.05 (black); *k*_2_/*k*_3_ = 0.25 (red); and *k*_2_/*k*_3_ = 0.5 (blue) and (f) *k*_3_ = 1 × 10^–5^ s^–1^ (blue); *k*_3_ = 1 × 10^–4^ s^–1^ (red); and *k*_3_ = 1 × 10^–3^ s^–1^ (black). (g–j) Flow velocity profiles among the source (red sphere) and drain (purple sphere), for increasing drain strengths from left to right. The red lines indicate the streamlines from the source to drain.

Marangoni flow patterns are pivotal in the design of our system to direct the filaments from the source toward selected drain droplets. We develop a model that predicts these patterns based on the rate constants involved in the depletion of amphiphiles from the air–water interface. Rather than modeling 3D Marangoni flow using finite element methods, we considered that a source which continuously releases amphiphile molecules to the air–water interface generates a radial Marangoni flow velocity *v*_source_ that is proportional to *r*^*n*^, with *r* being the distance from the source center (Figure 2a). In the literature, it has been shown that *n* ≈ −1 at high source concentration, when the spreading is dominated by dissolved surfactant molecules.^50^ In analogy, we hypothesized that when a surfactant-removing drain is included, the flow velocity *toward* the drain can also be approximated via *v*_drain_ ∼ *r*_drain_^–1^, with *r*_drain_ being the distance from the drain center (Figure 2b). Vectorial combination of *v*_source_, directed away from the source, and *v*_drain_, directed toward the drain, allows one to estimate the flow velocity and direction at any point at the air–water interface.

Next, we compute the flow velocity based on the amphiphile depletion rates, which depend on the rate constants involved. We assume that the amphiphile depletion from the air–water interface is predominantly driven by the Marangoni flow that transfers the air–water interface loaded with amphiphiles toward either the drain or the boundary of the flow zone, where the downward flow transfers the amphiphile toward the underlying bulk solution. Direct amphiphile depletion from the air–water interface to the underlying aqueous phase is anticipated to be minimal, as typical flow velocities of 20 μm s^–1^ imply only very minor surface tension gradients (see Supporting Discussion, Figures S1 and S2).

To simulate the amphiphile depletion rate, we revisited our kinetic model^42^ that describes how the surface tension depends on the rates of amphiphile release from the source droplet to the air–water interface (Φ_source_, in mol cm^–2^ s^–1^) and depletion from the air–water interface toward the drain droplet (Φ_drain_) and toward the underlying bulk aqueous phase (Φ_water_), Figure 2c. *A*_s_ equals the surface concentration of amphiphiles present in the source droplet and filaments, Γ is the surface concentration of amphiphiles adsorbed at the air–water interface, θ is the concentration of vacant adsorption sites at the air–water interface, *A*_d_ is the surface concentration of amphiphiles in the drain, and *A*_m_ is the concentration of amphiphiles dissolved in the underlying aqueous phase. Via the Frumkin isotherm, the surface tension γ can be calculated from Γ.^51^ The model predicts a rapid saturation of the interface when a source droplet is deposited. Upon deposition of the drain (i.e., changing the rate constant *k*_2_ = 0 to *k*_2_ > 0), the overall depletion rate increases from Φ_water_·*a* to (Φ_water_ + Φ_drain_)·*a*, with *a* being the area of the air–water interface (Figure 2d).

Departing from this kinetic model, which describes amphiphile depletion from the air–water interface as a homogeneous system, we now estimate the velocity profiles based on these depletion rates. At the boundary of the flow zone with radius *R*, each second, a ring-shaped air–water interface with an area of 2π*R*·*v*_source_(*R*)·1s (with *v*_source_ in mm s^–1^ and *v*_source_(*R*)·1s ≪ *R*) and amphiphile surface concentration Γ = 4.458 × 10^–10^ mol cm^–2^ is depleted from the air–water interface via the counterflow, as schematically shown in Figure 2a. Hence, *v*_source_(*R*) can be determined via Φ_water_·*a* = Γ·2π*R*·*v*_source_(*R*). Subsequently, the relation *v*_source_ ∼ *r*^–1^ allows us to determine *v*_source_(*r*). In analogy, the relation Φ_drain_·*a* = Γ·2π*R*_drain_·*v*_drain_(*R*_drain_), with *R*_drain_ being the radius of the drain droplet, allows determination of *v*_drain_(*R*_drain_). We anticipate that the flow profiles from the source to drain depend on the ratio between *k*_*2*_ and *k*_*3*_, that is, drain and bulk depletion rates, as predicted by the model. To estimate the values of rate constants *k*_2_ and *k*_3_ based on surface tension dynamics (which are experimentally accessible to validate our model), we simulate the change in surface tension Δγ for a solution of C_12_E_4_ (above the cmc) upon deposition of the drain. As shown in Figure 2e, Δγ increases with the ratio *k*_2_/*k*_3_, implying that for a stronger drain (i.e., larger value of *k*_2_), the amphiphile depletion from the air–water interface by the drain outcompetes the resupply of amphiphiles from the underlying aqueous phase. The rate at which the new steady state is established increases with *k*_3_ (Figure 2f). In Figure 2g–j, flow profiles are simulated for scenarios with no drain (i.e., *k*_2_ = 0) and in the presence of drains with increasing strength, which are positioned at a fixed distance from the source. The simulations highlight how both the flow intensity and the number of streamlines going from the source toward the drain increase upon increasing the strength of the drain (i.e., *k*_2_ = 0.05·*k*_3_; *k*_2_ = 0.25·*k*_3_; and *k*_2_ = 0.5·*k*_3_). Importantly, this approach is essentially different from solving the overall hydrodynamic problem with mass transfer by advection and diffusion in addition to the adsorption/desorption kinetics, and it considerably simplifies with respect to the third dimension. Together, these simulations show how the Marangoni flow pattern, which dictates the number of filaments that are transferred to the drain, can be predicted based on rate constants involved in amphiphile release and depletion; these, in turn, can be related to surface tension measurements.

### 3.2 Experimental Study of the Marangoni Flow Patterns Around Source and Drain Droplets

**Figure 3:**
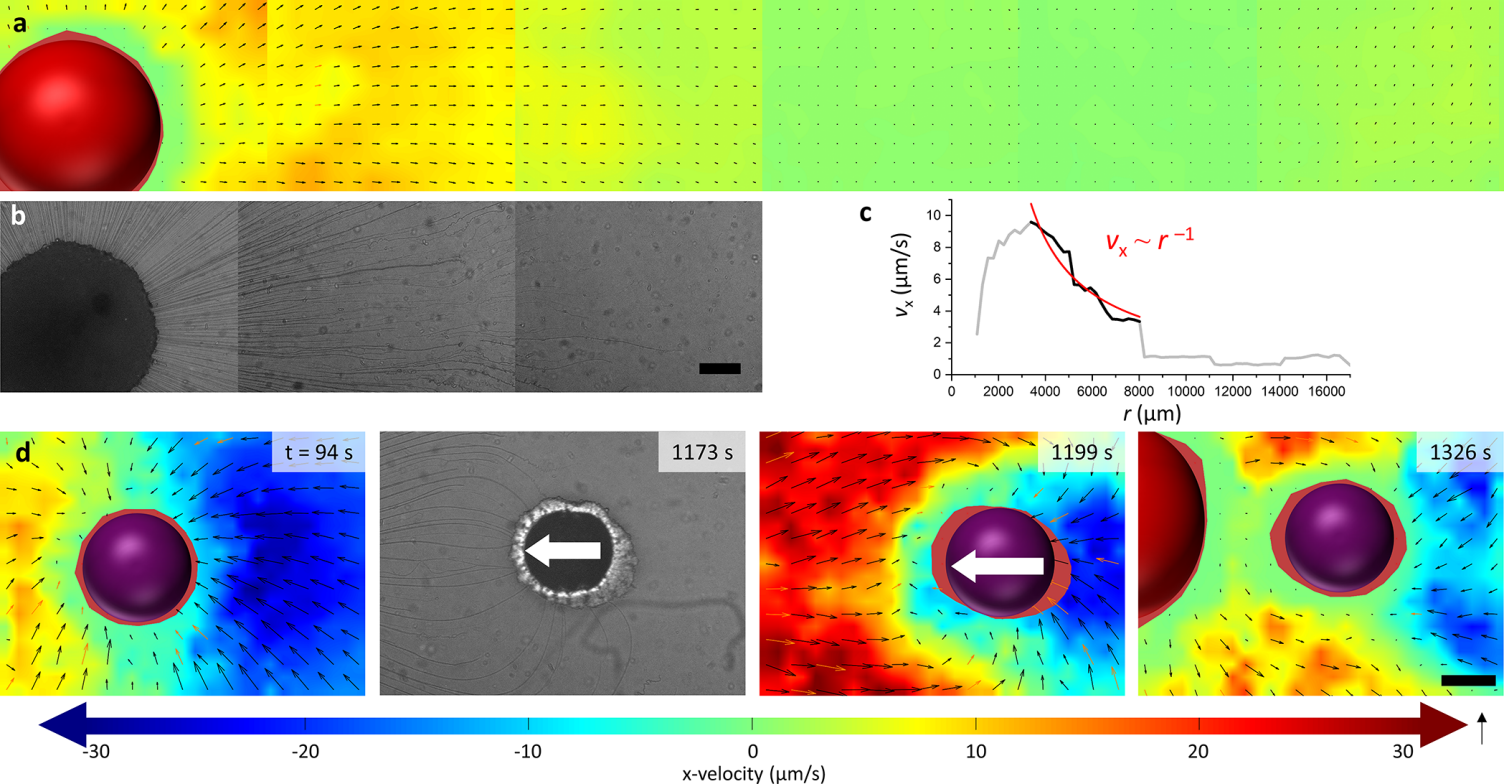
Visualization of the flow profiles surrounding source and drain droplets. (a) X-component of the Marangoni flow (*v*_x_) surrounding a C_12_E_4_ droplet. (b) Optical microscopy images corresponding to the first three panels of (a). (c) Average velocity *v*_x_ vs distance *r* from the center of the source droplet. *v*_x_ = 25,478/(*r* – 1014) was fitted with *R*^2^ = 0.912 in the region 3387 μm < *r* < 8014 μm. (d) Flow profile induced by a 10 wt % NaOleate/OA drain droplet shortly after deposition at *t* = 0 s (first panel), as it moves toward the source (third panel) and when it is stationary near the source (fourth panel). The second panel is a bright field image of the drain droplet. The scale bars represent 500 μm, and the arrow in the legend (bottom right) corresponds to a velocity of 41 μm s^–1^ in (a) and 30 μm s^–1^ in (d).

We studied the Marangoni flows experimentally using PIV. In our setup, the system was illuminated by a laser from the top, and fluorescence was emitted by polystyrene microparticles dispersed in the aqueous solution. This allowed us to acquire top view profiles of the flow close to the air–water interface, which were analyzed using the *PIVlab* plugin for MATLAB.^46^ Upon deposition of the C_12_E_4_ source droplet onto the air–water interface, a Marangoni flow emerges that is directed away from the source (Figure 3a,b and Movie S1). The velocity *v* reaches a maximum of 9 μm s^–1^ at a distance of *r* = 4 mm from the source droplet and then decreases gradually with the radius. We note that this wide-field PIV analysis comprises multiple panels that were acquired subsequently (see the Supporting Information), which may result in the steps at the seams of the panels (Figure 3c) In the range of *r* = 4–8 mm, the velocity can be described with the function *a*·(*r* + *b*)^−1^, indicating that *v* is proportional to *r*^–1^. Fluorescence microscopy experiments that provide side view profiles reveal a counterflow in the opposite direction a few millimeters under the air–water interface (Figure S3 and Movies S9, S10, and 11).

Next, we assessed the Marangoni flow upon deposition of a drain droplet that consists of 10 wt % sodium oleate in oleic acid (10% NaOleate/OA)—a drain that we previously reported to attract filaments and thereby participate in sustained self-organization.^42^ Right after deposition, a symmetric Marangoni flow profile emerges around the droplet, which is directed toward it (Figure 3d and Movie S2). Close to the drain, *v* increases up to approx. 20 μm s^–1^, indicating an enhanced Marangoni flow and therefore an increased surface tension gradient, which we attribute to the drain depleting surfactant from the air–water interface. Indeed, when a 10% NaOleate/OA drain is applied on an aqueous C_12_E_4_ solution, we observed an increase in surface tension (Figure S4). The outward Marangoni flow from the source would imply a drift of the drain droplet away from the source droplet. However, filaments that grow from the source are transferred by the Marangoni flow toward the drain, inducing its motion in the direction of the source upon attachment.^42^ Importantly, PIV reveals that at the sides of the drain, the flow profile is directed parallel to the movement of the drain droplet (Movie S3). We note that this flow profile is different from active swimmers that progress due to Marangoni propulsion. Here, literature reports show that the flow at the sides of the droplet is oriented opposite to the propagation of the droplet.^52−54^ Instead, our flow profile corresponds to the profile of a passive object moving through a fluid by an external force.^53,55^ Indeed, PIV analysis of the liquid inside moving drain droplets reveals the absence of internal Marangoni flow patterns at velocities that would be required to drive the motion of the drain droplet (see Figure S5 for further discussion and Movies S12 and 13). Together, these observations suggest that upon attachment of the filaments, an external force is applied that drives the motion of the drain toward the source.

### 3.3 Attraction of the Drain Droplet by Filaments via the Elastocapillary Effect

**Figure 4:**
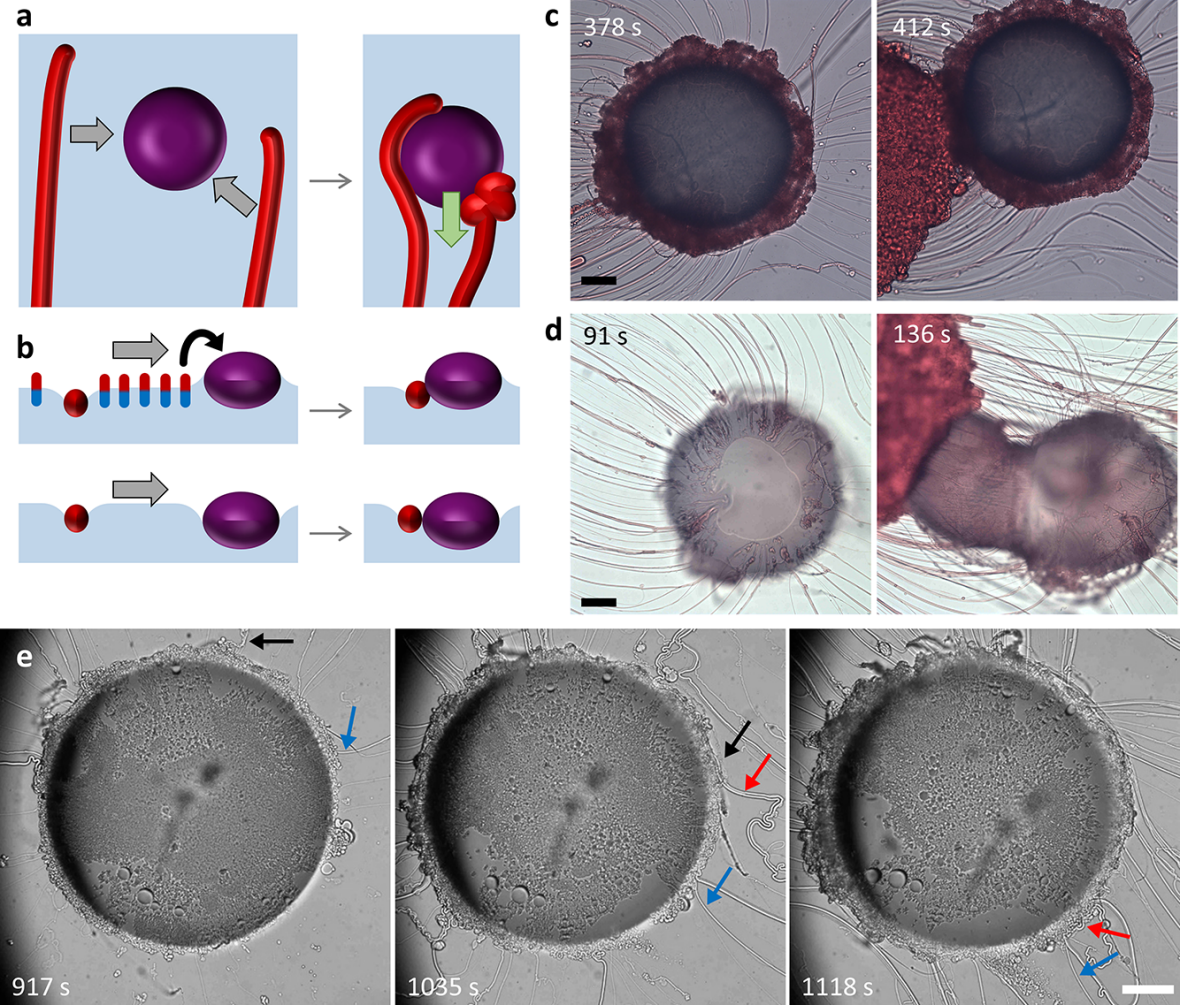
Elastocapillary wrapping of filaments around drain droplets. (a) Filament is bent by capillary adhesive forces. It partially wraps around the drain or coils up, drawing the drain toward the source. (b) If the menisci of filaments and drain have opposite sign (top), adhesion occurs only if the Marangoni flow brings them together. If their menisci have equal signs (bottom), the Cheerios effect pulls them together. (c,d) Optical microscopy images of 10% NaOleate/OA (c) and 10% C_12_E_4_/OA (d) drains before (left) and after (right) collision with a dyed source droplet. Filaments accumulate around the drains, while also being absorbed by the 10% C_12_E_4_/OA drain. (e) Optical microscopy images of filaments adhering to a 20% C_12_E_4_/OA drain droplet, deposited at *t* = 0 s. Inclusion of C_12_E_4_ reduces the absorption rate, such that adhering filaments remain intact. Three distinct filaments are indicated with black, blue, and red arrows as they wrap around the drain. The scale bars represent 200 μm.

The motion of the drain droplet toward the source prompted us to investigate how the filaments, which move along the Marangoni flow, interact with the drain upon arrival. The adhesion of the filaments to the drain and the subsequent motion of the drain back to the source could potentially be driven by elastocapillary effects. Recently, Prasath et al.^32^ demonstrated that a thin floating polydimethylsiloxane filament (diameter approx. 100 μm) wraps around a floating oil droplet, as capillary attraction provides the energy required to bend the filament. In our source–drain system, the tension on the filaments resulting from these forces can pull the drain toward the source, as the filaments wrap themselves around the drain or coil up into clusters (Figure 4a). The subsequent attraction and adhesion of filaments to the drain can potentially occur via two mechanisms (Figure 4b): (1) The Marangoni flow draws the filaments toward the drain, where, upon contact, elastocapillary effects cause the filaments to wrap around the drain droplets, which in turn get pulled toward the source. (2) The filaments and drain have an equally oriented meniscus at the air–water interface, and the filaments get attracted toward the drain via the Cheerios effect, where they adhere to the drain via capillary forces.

Both mechanisms imply that the filaments remain intact and adhere to the drain droplet, rather than being absorbed. To characterize the absorption or adhesion of filaments at the drain, experiments were conducted with C_12_E_4_ source droplets containing the dye Oil Red O. As shown in Figure 4c and the left panel of Movie S4, for a 10% NaOleate/OA drain, all filaments accumulate at the edge of the droplet. The red color does not penetrate into the opaque drain droplet, even when the drain and source collide, indicating that no material of the filaments is absorbed by the drain. This can be rationalized by the formation of a liquid crystalline film at the interface of the drain with the surrounding aqueous phase. Fd3m and L_2_ liquid crystalline phases have been reported in the literature when mixing 10% NaOleate/OA with an aqueous NaCl solution.^56^ Indeed, when a 10% NaOleate/OA solution was carefully applied on top of a NaAlg/NaCl solution, the interface was rapidly covered by a thin film. To investigate the relation between film formation and accumulation of filaments at the drain boundary, C_12_E_4_/OA drain droplets were studied. This type of drain does not form such a film when in contact with the NaAlg/NaCl solution. As shown in Figure 4d, as well as the right panel of Movie S4, a 10 v/v % C_12_E_4_ in OA drain absorbs filaments loaded with Oil red O, demonstrated by the coloring of the droplet interior. However, the absorption of filaments is incomplete, and some of the filaments accumulate or coil up into clusters at the edge of the droplet. To further suppress absorption, we increased the C_12_E_4_ content in the drain. For a 20 v/v % C_12_E_4_ in an OA drain droplet, we observed many filaments to wrap around the drain, rather than being absorbed; this can also be observed from the sideway approach of filaments toward the drain (Figure 4e and Movie S5). Subsequently, as these filaments accumulate at the drain, they also stick to each other and form a ring of filaments wrapped around the drain (Figure S6 and Movie S14).

**Figure 5:**
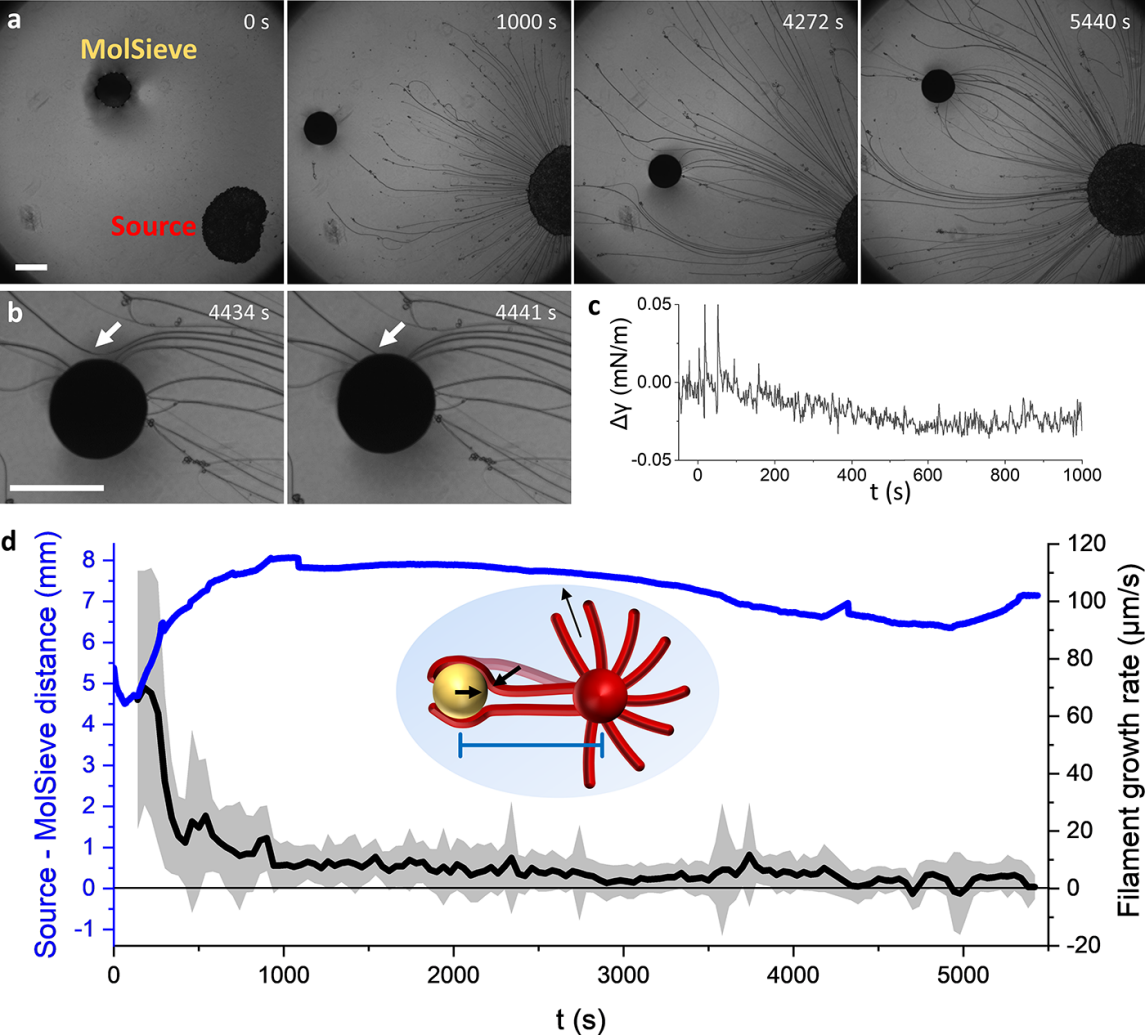
Attraction of a solid MolSieve by elastocapillary effects acting on the filaments. (a) Optical microscopy images of the self-organization of a floating C_12_E_4_ source and 4 Å MolSieve. (b) Filament (white arrow) approaches toward and attaches to the MolSieve. (c) Change in surface tension (Δγ) vs time upon the addition of a floating MolSieve at *t* = 0 s onto 17 mM NaCl and 0.52 mM C_12_E_4_ in MilliQ. The MolSieve displays no C_12_E_4_ uptake. (d) Center-to-center distance between the source and MolSieve (blue) and average growth rate of filaments versus time (black). Initially, the MolSieve is repelled from the source. When filaments attach to the MolSieve, the source—MolSieve distance remains constant, even though the filaments are still growing from the source, as schematically shown in the inset. Filament growth rate was approximated by tracking the movement of defects on filaments relative to the source (Supporting Information). The scale bars represent 1 mm.

To further assess the hypothesis of elastocapillary forces, we used solid spherical zeolite-based 4 Å molecular sieves (MolSieve) to attract the filaments, instead of oil-based drain droplets (Figure 5a,b and Movie S6). When deposited at an aqueous C_12_E_4_ solution, the Molsieve shows no depletion of C_12_E_4_, as evidenced from surface tension measurements (Figure 5c). When a MolSieve is deposited on an air–water interface at which a source droplet is already present, it is observed to move away from the source, driven by the outward Marangoni flow from the source droplet. However, we observe that small filament fragments are attracted to the MolSieve, and at approx. 1000 s, the first filaments connect to it, such that the MolSieve is kept in position despite the outward Marangoni flow, the presence of which is evidenced by the filaments that are still growing form the source (Figure 5d).

**Figure 6:**
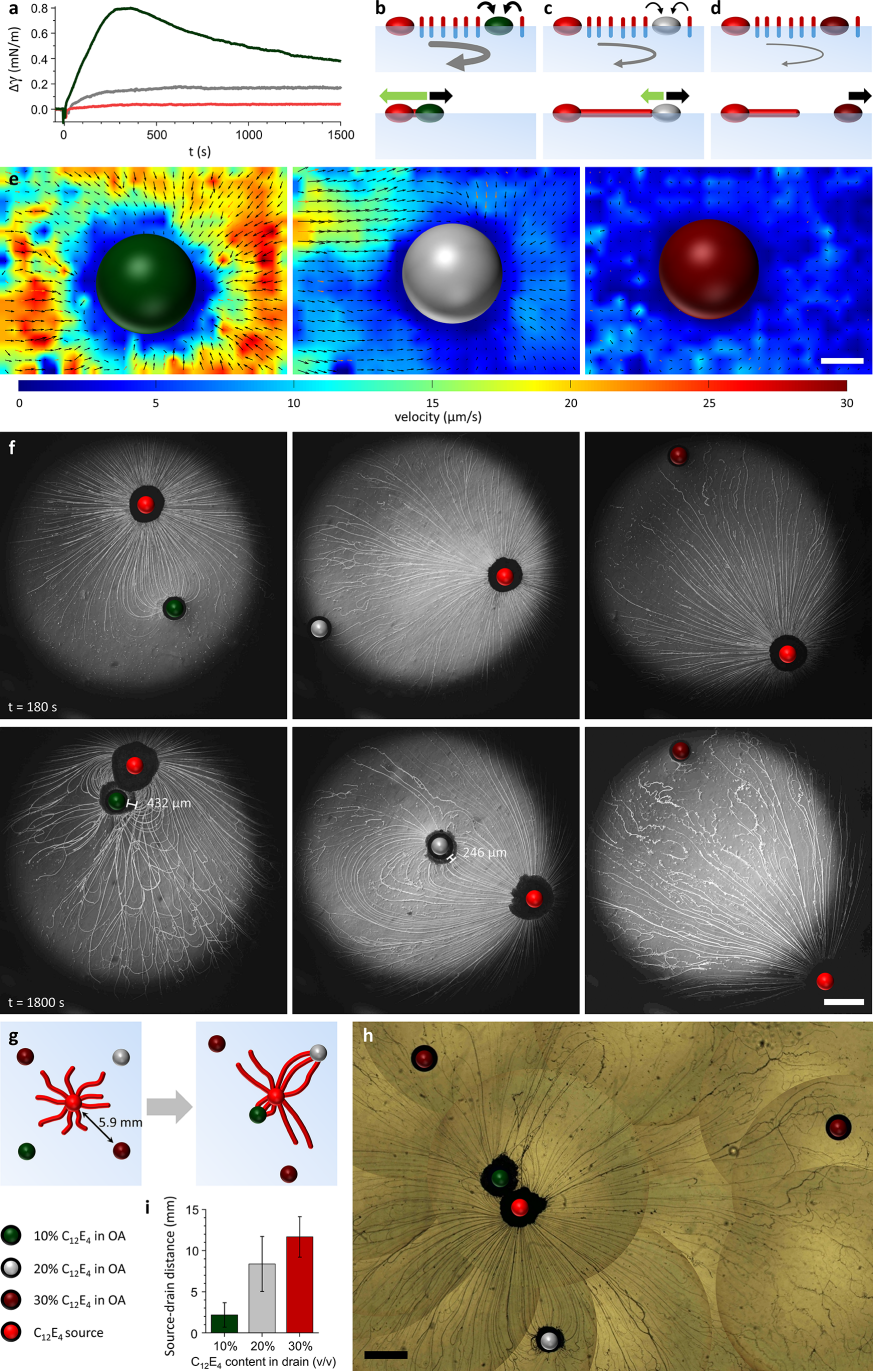
C_12_E_4_/OA drains with tunable depletion rate allow for selective attraction. (a) Change in surface tension (Δγ) vs time, upon deposition of an OA drain with 10% (dark green), 20% (gray), or 30% (v/v) (dark red) C_12_E_4_ on 17 mM NaCl and 0.52 mM C_12_E_4_ in MilliQ. (b–d) Depletion of amphiphiles by the drain generates a Marangoni flow (black arrow) which not only repels the drain but also carries filaments toward it that provide attractive forces (green arrow) which overcome (b) or balance (c) the flow. If the Marangoni flow is too weak to bring filaments to the drain (d), the drain is repelled. (e,f) Flow profile surrounding stationary C_12_E_4_/OA droplets near a C_12_E_4_ source (e), and optical microscopy images of their self-organization (f). From left to right: 10% C_12_E_4_/OA, 20% C_12_E_4_/OA, and 30% C_12_E_4_/OA. (g) When droplets are deposited as displayed, their differences in C_12_E_4_ depletion rate impact their attraction toward the source. (h) Optical microscopy experiment corresponding to (g). The images were acquired 20 min after droplet deposition. (i) Average center-to-center distance between the source and drain for OA-based drain droplets with different C_12_E_4_ contents (*n* = 5 separate experiments). The scale bar in (e) represents 500 μm; the scale bars in (f) and (h) represent 2 mm.

The MolSieve’s inability to deplete C_12_E_4_—and thereby induce Marangoni flow toward itself—suggests that the attraction of filaments toward the MolSieve is driven by the Cheerios effect. The MolSieve has a negative meniscus at the air–water interface: it moves toward the Petri dish wall when the aqueous solution forms a negative meniscus and away from a Petri dish wall with a positive meniscus. Thereby, the attraction of filaments to the MolSieve implies that the filaments have a negative meniscus as well. Gratifyingly, for a 30% C_12_E_4_/OA drain, which does not deplete C_12_E_4_ from the air–water interface and consequently does not attract a Marangoni flow, but has a positive meniscus, we observed no attraction of filaments (Figure 6f).

Together, these observations show that the motion of filaments toward the drain can occur via (1) the Marangoni flow and (2) the Cheerios effect, which requires the meniscus of the drain to be of equal sign as the filaments (i.e., negative). When the filaments are brought in close proximity to the drain via the Marangoni flow, capillary adhesion of filaments to the drain can even occur with a different sign of the drain and filament menisci.^37,57,58^ However, in the absence of the Marangoni flow, the unequal menisci will cause repulsion between filaments and drain. Thereby, mechanism (1) opens up potential for a selective transfer of filaments toward the drains with a strong surfactant depletion (Φ_drain_), as simulated in Figure 2, followed by attraction of the drains toward the source via the elastocapillary effect.

### 3.4 Selective Attraction of Drain Droplets by the Filaments

Now that the role of the elastocapillary effect has been demonstrated, we attempt to display the unique behavior enabled through the connectivity of the filaments by establishing a source droplet that selectively attracts floating drain droplets, based on their chemical composition. In the simulations, we established that the flow that carries filaments to the drain depends on the rate at which the drain depletes C_12_E_4_ from the air–water interface (Φ_drain_, determined by rate constant *k*_2_, Figure 2). In our experiments, we therefore used three different drain droplets (10% C_12_E_4_/OA, 20% C_12_E_4_/OA, and 30% C_12_E_4_/OA) to tune the strength of attraction. Surface tension measurements show how the inclusion of increasing amounts of C_12_E_4_ decreases the rate at which the drain depletes C_12_E_4_ from the air–water interface (Figure 6a). Indeed, the increase in surface tension Δγ that was observed matches with the simulated Δγ for *k*_2_ = 0.5·*k*_3_ (10% C_12_E_4_/OA), *k*_2_ = 0.25·*k*_3_ (20% C_12_E_4_/OA), and *k*_2_ = 0.05·*k*_3_ (30% C_12_E_4_/OA). We expect the Marangoni effect generated by the presence of these droplets to vary in strength based on their depletion rate (Figure 6b,c), to the point where the flow is not strong enough to attract filaments toward the drain (Figure 6d). PIV measurements confirm that the flow velocity toward the drain decreases upon increasing the C_12_E_4_ content in the drain droplet (Figure 6e and Movie S7). Next, optical microscopy shows how these drains interact with the floating filaments. Corresponding with the streamlines simulated in Figure 2, a larger C_12_E_4_ content in the drain results in a smaller number of filaments being attracted, as can be observed from the thickness of the filament clusters surrounding these drains (Figure 6f and Movie S8). The 10% C_12_E_4_/OA drain is tethered to a large number of filaments, and these filaments coil up into a corona around the drain droplet, exerting an average force of approximately *F* = 6·π·μ·*R*_drain_·*v*_drain_ = 2 nN to draw the drain close to the source (with viscosity μ = 5 mPa s, *R*_drain_ = 0.5 mm, and *v*_drain_ = 39 μm s^–1^ between *t* = 150–250 s, Movie S8). We note that the velocity of the drain increases as the number of filaments tethered to the drain increases over time. As a consequence, the lower amount of filaments that is drawn by the Marangoni flow toward the 20% C_12_E_4_/OA drain results in a slower attraction, and this drain remains at a distance from the source even after 30 min. The 30% C_12_E_4_/OA drain is instead repelled by the Marangoni flow that arises from the source, while no filaments connect to provide an attractive force. Finally, when a C_12_E_4_ source droplet and four drain droplets with different C_12_E_4_ contents are simultaneously deposited at equidistant positions at the air–water interface, these differences in source–drain interaction are translated into selective self-organization of the droplets after 15–27 min, featuring source–drain distances that correlate with the C_12_E_4_ content of the individual drain droplets (Figure 6g–i and Figure S7).

## 4 Conclusions

We unraveled how Marangoni and elastocapillary effects direct the self-organization of floating droplets and amphiphile filaments at air–water interfaces. First, the release of C_12_E_4_ amphiphiles from an amphiphile source droplet drives the Marangoni flow in the aqueous solution, which was successfully visualized through PIV analysis. If a drain is then deposited, the uptake of amphiphile molecules from the air–water interface by the drain directs the Marangoni flow toward it. The drain thereby attracts the amphiphile filaments that grow from the source, with higher amphiphile uptake corresponding to stronger attraction, as predicted by Marangoni flow patterns in our model. Subsequently, the filaments that come into contact with the drain can exert an attractive force via elastocapillary effects, enabling a mechanism for selective attraction in self-organization of floating droplets.

We note that the emergence of gradients offers a general design principle to establish spatial differentiation in self-organization processes. The release of surface-active compounds from floating objects has been exploited to generate Marangoni flows that drive their mutual repulsion and thereby the self-organization.^59^ However, although such a Marangoni flow would result in repulsion of all floating objects, our amphiphile filaments—which connect only to some of the floating droplets—can exert selective forces by which the droplets are attracted. Together, these findings were employed to create a self-organizing system in which a source droplet differentiates between drain droplets based on their composition, by selectively forming connections between elements which, through said connections, establish self-organization. Understanding the physicochemical phenomena at play in interfacial source–drain systems paves the way for the construction of out-of-equilibrium self-organizing networks with dynamic connectivity, for example, by including dissipative source and/or drain elements, larger numbers of droplets, and other mechanisms that lead to pattern formation. In doing so, we start to bridge the gap between synthetic and biological systems, ultimately distilling their complex functionalities into simple building blocks.

## Supporting Information Available

Additional experimental details, details on the simulations of the Marangoni flow patterns, additional simulations, tensiometry data, microscopy images, and PIV analysis (PDF)
Source flow profile (AVI)
Stationary drain inducing a roughly symmetrical flow (AVI)
PIV analysis of flow profile around the drain that is attached to filaments (AVI)
Accumulation of filaments at the edge of the droplet and absorption of filaments loaded with Oil red O (AVI)
Filament wrapping (AVI)
MolSieve self-organization (MP4)
PIV analysis of the flow profile around the C_12_E_4_/OA drains (AVI)
Thickness of the filament clusters surrounding the drains (AVI)
Side-view PIV analysis of the solution (AVI)
PIV at the air–water interface (AVI)
PIV at z = −1mm under the air–water interface (AVI)
PIV analysis of the flow profile inside a 10% NaOleate/OA drain (AVI)
PIV analysis of the flow profile inside a 10% C_12_E_4_/OA drain (AVI)
Filament ring around a 20% C_12_E_4_/OA drain (AVI)

## Supplementary Material

la2c01241_si_001.pdf

la2c01241_si_002.avi

la2c01241_si_003.avi

la2c01241_si_004.avi

la2c01241_si_005.avi

la2c01241_si_006.avi

la2c01241_si_007.mp4

la2c01241_si_008.avi

la2c01241_si_009.avi

la2c01241_si_010.avi

la2c01241_si_011.avi

la2c01241_si_012.avi

la2c01241_si_013.avi

la2c01241_si_014.avi

la2c01241_si_015.avi
