# Supplementary material for: Self-Organization Emerging from Marangoni and Elastocapillary Effects Directed by Amphiphile Filament Connections
Source: Langmuir. 2022 Aug 25;38(35):10799–809. doi: 10.1021/acs.langmuir.2c01241 (PMC9454263; doi:10.1021/acs.langmuir.2c01241)
Supplement: Supplementary file 1 — la2c01241_si_001.pdf [file la2c01241_si_001.pdf]

Self-organization emerging from Marangoni and elastocapillary effects directed by amphiphile filament connections

*Mitch Winkens, Peter A. Korevaar**

Institute for Molecules and Materials, Radboud University, Heyendaalseweg 135, 6525 AJ Nijmegen, The Netherlands

e-mail: p.korevaar@science.ru.nl

Supporting Information

Content

1. Additional experimental details

2. Details on the simulations of the Marangoni flow patterns

3. Supporting Figures

4. Description of Supporting Movies

5. References

1. Additional experimental details

Instruments and settings

Optical microscopy images and movies were acquired at 1 fps with an Olympus IX73 dark field inverted microscope equipped with a Point Grey Grasshopper3 camera, controlled using FlyCapture software. An Olympus Plan Apo N 1.25x 0.04 NA objective was used for the optical microscopy experiments shown in Figures 6f and h, S6 (first three panels) and S7, as well as Movies S8 and S14 (first half). Images shown in Figure 5 were recorded using an Olympus Plan N 2x 0.06 NA objective. An Olympus UPlan FL 10x 0.30 NA objective was used in Figure 4 and Movies S4-5, and an Olympus LUCPlan FL N 20x 0.45 NA objective was used to record the rightmost panel of Figure S6 and the second half of Movie S14.

Top-view fluorescence microscopy images and movies were acquired using a Leica DM2500 upright microscope equipped with a DFC 7000T camera, controlled using LasX software. All such experiments were recorded using a Leica HCX Plan FL 2.5x 0.07 NA objective, except for those shown in Figure S3c-d, which were recorded with a Leica HC PL Fluotar Ph1 10x 0.30 NA objective. Samples were illuminated using a HXP R 120 W/45C Osram lamp, combined with an excitation filter of 515-560 nm and an emission filter of 590 nm. Images were recorded with an exposure time of 450 ms at 1.02 fps for Figures 3, 6e (middle panel), S5a, Movies S1-3, and the middle panel of Movie S7; with an exposure time of 200 ms at 2.06 fps for Figure 6e (left and right panel), S3c-d, the left and right panels of Movie S7, and Movies S10-11; and with 50 ms exposure time at 5.41 fps for Figures S5d-e and Movies S12-13.

Side-view fluorescence microscopy images and movies were acquired using a Dino-Lite AM4515ZT microscope at 50x magnification, controlled by DinoCapture 2.0 software and illuminated using a 120 W mercury lamp combined with an emission filter of 599/59 nm.

The surface tension kinetics were investigated using a KSV Instruments LTD surface tensiometer, measuring the force exerted by the air-water meniscus on a platinum Wilhelmy plate (19.62 mm x 10 mm). Before each measurement, the Wilhelmy plate was cleaned using ethanol, exposed to an open flame until red hot, and equilibrated in air.

Microscopy experiments and PIV analysis.

In the experiments shown in Figures 3a-b and Movie S1, 1.0 µL $C_{12}E_4$ was deposited onto 5.5 mL medium solution. After waiting for approximately 11 minutes, movies were then recorded at six positions, starting at 15 mm from the source, then moving 3 mm closer to the source with each window over the course of approximately 500 seconds. For Figure 3a, PIV analysis was performed using PIVlab[1] on the six individual panels displayed in Movie S1. A Wiener2 denoise filter was applied with a window of 3 pixels, as well as the auto contrast stretch function. Fast Fourier transform (FFT) analysis was then

performed at extreme correlation quality[2] with an interrogation area of 256 pixels, with the step size equal to 50% of the interrogation area. The velocity magnitude was then calculated for each frame, and any frames with velocity magnitude deviating from the average by more than 2 standard deviations was removed from the analysis. The flow fields displayed in Figure 3a were calculated from the mean velocity fields of the remaining frames, followed by vector validation with a standard deviation filter threshold and local median filter threshold set to 3. Deviating vectors were replaced by interpolating the surrounding vectors, and are displayed in orange. Finally, the velocity values displayed in Figure 3c were calculated by averaging each vertical column of vectors displayed in Figure 3a. Fitting of the function $a / (r - b)$ was done using the simple fit function of Origin 2019b for 3387 μm < r < 8014 μm, confined by the r-coordinate at which the maximum velocity was found and that at which the velocity started decaying sharply.

In the experiment shown in Figures 3d and S5a and Movies S2-3, 1.0 μL $C_{12}E_4$ was deposited onto 5.5 mL medium solution, followed by 1.0 μL 10% NaOleate in OA drain solution after 4 minutes. For the first panel, PIV analysis was performed on frames 26-29 of Movie S2, while the third and fourth panels correspond to frames 25-28 and 175-178 of Movie S3, respectively. A Wiener2 denoise filter was applied with a window of 3 pixels, as well as the auto contrast stretch function. Two-pass FFT analysis was then performed at extreme correlation quality with an interrogation area of 256 pixels for the first pass and 128 pixels for the second pass, with the step size equal to 50% of the interrogation area. The average flow fields were then calculated from each set of analyzed frames, and vector validation was applied with a standard deviation filter threshold and local median filter threshold set to 3. Only one out of every four vectors is displayed in Figure 3d to improve the clarity of the PIV image.

In the experiments shown in Figures 4c-d and Movie S4, 1.0 μL 5.5 mg/mL Oil red O in $C_{12}E_4$ was deposited onto 5.5 mL medium solution, followed by 1.0 μL drain solution (10% NaOleate in OA and 10% $C_{12}E_4$ in OA for Figure 4c and Figure 4d, respectively). The setup was covered with a glass petri dish after deposition of the drain droplet in order to suppress evaporation.

In the experiment shown in Figure 4e and Movie S5, 1.0 μL 20% $C_{12}E_4$/OA was deposited onto 5.5 mL medium solution, followed by 1.0 μL $C_{12}E_4$, and the setup was covered using a glass petri dish in order to suppress evaporation.

In the experiment shown in Figure 5a-b and Movie S6, 1.0 μL $C_{12}E_4$ was deposited onto 4 mL medium solution, followed after 150 seconds by a 4Å 4-8 mesh molecular sieve (activated at 100 °C) deposited using tweezers. For Figure 5d, source-drain distance was calculated based on the coordinates of the center of the drain and source for every analyzed frame. Using the same method, the outward movement of defects was calculated relative to the center of the source droplet, and was assumed to be representative for the growth rate of the filaments.

The positions of the free-floating source droplet and 4Å molecular sieve, as well as those of defects on filaments, were analyzed over time using the TrackMate algorithm for Fiji.[3,4] Before analysis, frames 1-159 were cut from the movie, as no filament growth was observed. In order to further reduce the file size of the original movie, 3 out of every 4 frames were then removed, and the top 84 pixels were removed from all images. Furthermore, the black-and-white values of images were inverted. Before determining the position trace of the MolSieve, a Laplacian of Gaussian (LoG) filter was applied. Objects of diameter 1.1 mm were then detected, with thresholding factor 0.01. Duplicate objects, i.e. two objects detected in the same frame, were deleted manually. The position trace of the source was determined after applying a downsample LoG filter, with downsampling factor 4. Objects of diameter 2.6 mm where then detected, with thresholding factor 0.02. Duplicate objects were deleted manually. Finally, to probe the growth of the filaments, the position traces of defects at the filaments were determined after applying a LoG filter, by detecting objects with a diameter of 0.06 mm with thresholding factor of 2.5. Both linking and gap-closing distance were set at 0.4 mm, with a maximum gap of 5 frames. Finally, all traces containing less than 50 points were deleted. As insufficient defects were detected for frame 1-70 with these parameters, an additional TrackMate analysis was done on these frames with lower sensitivity: 0.05 mm diameter with the threshold at 0.66 and a maximum pixel intensity of 216. Both linking and gap-closing distance were set at 0.2 mm, with a maximum gap of 5 frames. Finally, all traces containing less than 10 points were omitted from the analysis.

Data from the following frames was removed from the analysis, due to movement of the stage and/or erroneous detection of the source coordinates: 72-73, 111-112, 231, 271-272, 1041, 1044, 1047, 1048, 1080, 1235, 1331. To reduce variance, each point on the graph represents a period of 40s (20s before the point until 19s after the point). For each defect detected during this window, the mean velocity during this period is determined, and the weighted average velocity of all defects is then calculated, with the weight for each defect equal to the number of frames during which it remains in view. The displayed standard error was calculated in the same way.

In the experiments shown in Figures 6e-f and S6, as well as Movies S7, S8 and S14, 1.0 μL $C_{12}E_4$ was deposited onto 5.5 mL medium solution, followed by 1.0 μL drain solution (For Figures 6e-f and Movies S7 and S8: 10% (v/v) $C_{12}E_4$ in OA in the left panel; 20% in the middle panel; and 30% in the right panel). Figure S6 and Movie S14 correspond to the same experiment as the one shown in the middle panel of Figure 6f and Movie S8. In the optical microscopy experiments, the setup was covered with a glass petri dish after deposition of the drain droplet in order to suppress evaporation. In the experiment displayed in the left panel of Figure 6f and of Movie S8, the petri dish is removed again at t = 1826 s, likely causing the observed disruption of the source droplet. In the experiment displayed in Figure S6 and Movie S14, a similar disruption of the source is seen after the petri dish is removed at t = 8698 s.

PIV analysis was performed for the experiments displayed in Figure 6e and Movie S7. For the left panel of Figure 6e, frames 43-46 of the leftmost panel in Movie S7 were analyzed; for the middle panel of Figure 6e, frames 14-17 of the middle panel of Movie S7; and for the right panel of Figure 6e, frames 72-78 of the rightmost panel of Movie S7 were analyzed. On these frames, a Wiener2 denoise filter was applied with a window of 3 pixels, as well as the auto contrast stretch function. Two-pass FFT analysis was then performed at extreme correlation quality with an interrogation area of 256 pixels for the first pass and 128 pixels for the second pass, with the step size equal to 50% of the interrogation area. The average flow fields were then calculated from each set of analyzed frames, and vector validation was applied with a standard deviation filter threshold and local median filter threshold set to 3.

For the experiments displayed in Figures 6h and S7, a CappAero384 64-channel pipette (distance between adjacent tips = 4.2 mm) was used to deposit onto 5.5 mL medium solution a droplet of 1.0 µL $C_{12}E_4$, simultaneously with 1.0 µL 10% $C_{12}E_4$ in OA, in which 10 mg/mL solvent green 3 was dissolved, 1.0 µL 20% $C_{12}E_4$ in OA, and 2x1.0 µL 30% $C_{12}E_4$ in OA, in which 3.0 mg/mL Oil red O was dissolved, in the following 3x3 pattern:

30% $C_{12}E_4$ in OA		20% $C_{12}E_4$ in OA
	$C_{12}E_4$	
10% $C_{12}E_4$ in OA		30% $C_{12}E_4$ in OA

The setups were then covered using a glass petri dish. The composed microscopy images were assembled manually from multiple frames of the recording taken between 15 and 27 min. after deposition of the droplets. Center-to-center distances of drain droplets to the source were measured manually and averaged over data acquired from 5 separate experiments. Images were acquired using an Olympus Plan Apo N 1.25x 0.04 NA objective and a frame rate of 1 fps.

In the experiment shown in Figure S3b and Movie S9, 1.0 µL $C_{12}E_4$ was deposited onto 5.5 mL medium solution. To create Figure S3b, pixel intensities of frames 69-83 of Movie S9 were averaged using version 1.53 of Fiji,[4] followed by contrast enhancement such that 5% of all pixels were saturated.

In the experiment displayed in Figures S3c-d and Movies S10-11, 1.0 µL $C_{12}E_4$ was deposited onto 5.5 mL medium solution. Flow velocity profiles were then measured at 5 mm distance from the source droplet, first at the interface at t = 656.5 - 685.1 s after deposition of the source, and then 1 mm below the interface at t = 690.0 - 718.6 s, for Figure S3c / Movie S10 and Figure S3d / Movie S11, respectively. PIV analysis was then performed on the frames displayed in Movies S10 and S11, with the region of interest set from $200 \leq x \leq 1824$ and $337 \leq y \leq 1389$ in order to remove particularly large floating fragments from the analyzed area. Contrast-limited adaptive histogram equalization was applied with window size set to 40 pixels, as well as a Wiener2 denoise filter with a window of 3 pixels, and the auto contrast stretch function. Two-pass FFT analysis was then performed at extreme correlation quality with an interrogation area of 256 pixels for the first pass and 128 pixels for the second pass, with the

step size equal to 50% of the interrogation area. The average vector field was then calculated over the course of both movies, and vector validation was applied with a standard deviation filter threshold and local median filter threshold set to 3. Finally, the average u-component of velocity (directed away from the source droplet) was calculated over both these resulting vector fields, yielding an average u-velocity of 18 μm/s for Movie S10 and 80 nm/s for Movie S11. Only one out of every four vectors is displayed in the final images to improve visibility.

In the experiments displayed in Figures S5d-e and Movies S12-13, 1.0 μL $C_{12}E_4$ was deposited onto 5.5 mL medium solution, followed by 1.0 μL drain solution (10% NaOleate in OA and 10% $C_{12}E_4$ in OA for Figure S5d and S5e, respectively). The frames displayed in the left panels of Movies S12 and S13 were then manipulated using version 1.53 of Fiji[4] to allow for automatic detection of the drain droplet in each frame. Images where converted to 8-bit, then the 'smooth' function was applied four times. The 'find edges' function was then applied, and finally the images were converted to binary using the 'threshold' function, with boundaries calculated automatically for each frame. The resulting images were analyzed using the 'Hough circle transform' plugin in order to detect the position of the drain droplet in each frame. The minimum search radius was set to 315 pixels, the maximum search radius to 320 pixels (N.B. the resolution of the original images was 1920 x 1440 pixels), and the radius search increment to 1 pixel. The maximum number of circles to be found was set to 1, with a Hough score threshold of 0.1 and transform resolution set to 1000. Local search was enabled with local radius search bandwidth and local search radius for position of next centroid both set to 100. Using the results of this analysis, each frame in the original movies was cropped to a 660 x 660-pixel window centered on the corresponding drain coordinate. This resulted in the movies displayed in the right panels of Movies S12-13, in which the position of the (moving) drain droplets is fixed. PIV analysis was then performed on these movies. A Wiener2 denoise filter was applied with a window of 3 pixels, as well as the auto contrast stretch function. FFT analysis was then performed at extreme correlation quality with an interrogation area of 64 pixels, with the step size equal to 50% of the interrogation area. The average flow fields were then calculated from both movies, and vector validation was applied with a standard deviation filter threshold and local median filter threshold set to 3.

2. Details on the simulations of the Marangoni flow patterns

Kinetic model to simulate surface tension kinetics. The kinetic model that predicts the surface tension, which we use to predict the Marangoni flow patterns amongst source and drain droplets (*vide infra*), is an extension of the model that we previously published.[5] We start off with this kinetic model, which describes the amphiphile depletion from the air-water interface as a homogeneous system.

Based on the reaction equations presented in **Figure 2c**, we defined the following rate equations for the surface concentration of amphiphiles in the source droplet and filaments A_s (in mol cm^{-2}):

(1) $$\frac{dA_s}{dt} = -k_1 \cdot A_s \cdot \theta \,;$$

N.B.: A_s is the concentration of amphiphiles in the source droplet and filaments, defined as if they are homogeneously distributed over the air-water interface. Later, we will use the depletion rate of amphiphiles – predicted by the kinetic model – to simulate the flow patterns between source and drain droplets with defined locations at the air-water interface.

Next, we define the rate equation for the surface concentration of the vacant sites at the interface θ (in mol cm^{-2}):

(2) $$\frac{d\theta}{dt} = -k_1 \cdot A_s \cdot \theta + k_3 \cdot \Gamma - k_{-3} \cdot A_m \cdot \theta + k_2 \cdot \Gamma \,;$$

the surface concentration of amphiphiles at the air-water interface Γ (in mol cm^{-2}):

(3) $$\frac{d\Gamma}{dt} = k_1 \cdot A_s \cdot \theta - k_3 \cdot \Gamma + k_{-3} \cdot A_m \cdot \theta - k_2 \cdot \Gamma \,;$$

and the concentration of amphiphiles in the underlying aqueous phase A_m (in mol cm^{-3}):

(4) $$\frac{dA_m}{dt} = \alpha \cdot k_3 \cdot \Gamma - \alpha \cdot k_{-3} \cdot A_m \cdot \theta.$$

Here, α represents the ratio of air-water interface per aqueous phase volume (in cm^2/cm^3).

The release rate of amphiphiles from the source droplet and filaments to the air-water interface Φ_{source} (in mol cm^{-2} s^{-1}) can be described by:

(5) $$\Phi_{\text{source}} = k_1 \cdot A_s \cdot \theta \,;$$

the depletion rate of the amphiphile towards the aqueous phase Φ_{water} (in mol cm^{-2} s^{-1}) by:

(6) $$\Phi_{\text{water}} = k_3 \cdot \Gamma - k_{-3} \cdot A_m \cdot \theta \,;$$

and the depletion rate towards the drain Φ_{drain} (in mol cm^{-2} s^{-1}) via:

(7) $\Phi_{\text{drain}} = k_2 \cdot \Gamma$.

To compute the surface tension γ (in mN m^{-1}) based on Γ, we used the Frumkin isotherm, as reported by Hsu et al.[6]

(8) $\gamma = \gamma_0 + 10^7 \cdot \Gamma_\infty RT\left(ln\left(1-\frac{\Gamma}{\Gamma_\infty}\right) - \frac{K}{2}\left(\frac{\Gamma}{\Gamma_\infty}\right)^2\right)$.

Here, γ_0 represents the surface tension of the air-water interface (72 mN m^{-1} for pure water); R the gas constant, T the temperature (T = 293 K), K the adsorption cooperativity factor (K = 1.875) and Γ_∞ the maximum surface concentration ($\Gamma_\infty = 4.663 \cdot 10^{-10}$ mol cm^{-2}); these values are reported by Hsu et al.[6]

Determining the equilibrium constant for the distribution of amphiphile between the air-water interface and the aqueous solution (K_3).

The relation between Γ and A_m can be determined via the Frumkin adsorption isotherm, as reported by Hsu et al.[6]:

(9) $\frac{\Gamma}{\Gamma_\infty} = x = \frac{A_\text{m}}{A_\text{m} + A\exp(Kx)}$,

with $A = 3.521 \cdot 10^{-10}$ mol cm^{-3} (ref. 6). Next, close to saturation of the air-water interface, where $\Gamma \approx \Gamma_\infty$, eq. 9 can be approximated by:

(10) $\frac{\Gamma}{\Gamma_\infty} = \frac{A_\text{m}}{A_\text{m} + A\exp(K)}$,

and with $K_3 = A \cdot \exp(K) = 2.296 \cdot 10^{-9}$ mol cm^{-3},

(11) $\Gamma = \frac{A_\text{m} \cdot \Gamma_\infty}{K_3 + A_\text{m}}$.

We note that eq. 11 can also be derived from reaction equation 3 in **Figure 2c** that provides the following equation for the equilibrium constant K_3:

(12) $K_3 = \frac{k_3}{k_{-3}} = \frac{A_\text{m} \cdot \theta}{\Gamma} = \frac{A_\text{m} \cdot (\Gamma_\infty - \Gamma)}{\Gamma}$.

Combining eq. 11 with eq. 8 allows one to plot the surface tension γ *vs*. total amphiphile concentration [$C_{12}E_4$], assuming that if [$C_{12}E_4$] > critical micelle concentration (CMC); A_m = CMC and hence γ does not decrease beyond the CMC. As shown in **Figure S1**, with $K_3 = 2.296 \cdot 10^{-9}$ mol cm^{-3}, the γ *vs*. A_m - plot matches with the experimentally reported surface tension of $C_{12}E_4$ of 27 mN m^{-1} at its CMC of $5 \cdot 10^{-8}$ mol cm^{-3}. With $A_\text{m} = 5 \cdot 10^{-8}$ mol cm^{-3}, eq. 11 yields $\Gamma_{\text{CMC}} = 4.458 \cdot 10^{-10}$ mol cm^{-2}.

To simulate the depletion rate in **Figure 2d**, we defined the starting values for A_s, θ, Γ and A_m. Initially, all amphiphile molecules are present in the source droplet, implying that $A_\text{s}(t = 0)$ can be derived from

the volume of the amphiphile source droplet and the area of the air-water interface; $\Gamma(t = 0) = 0$ and $A_m(t = 0) = 0$. The concentration of vacant positions available at the air-water interface $\theta(t = 0)$ was derived from the equilibrium density of amphiphiles present at the air-water interface at the critical micelle concentration ($\Gamma_{CMC} = 4.458\cdot10^{-10}$ mol cm^{-2}, *vide supra*). The simulation in **Figure 2d** was performed by solving the system of differential equations (1–4) using Matlab (R2017a, *ode15s solver*), with $k_1 = 1\cdot10^8$ cm^2 mol^{-1} s^{-1}; $k_3 = 1\cdot10^{-4}$ s^{-1}; $\alpha = 2$ cm^2/cm^3; and $A_s(t = 0) = 2.7\cdot10^{-7}$ mol cm^{-2}. The value of k_1 is chosen such that the model predicts a rapid decline in surface tension when a source droplet is applied at the air-water interface, in agreement with simulations reported in ref. 5 (van der Weijden et al.). The value of k_3 is chosen such that the model predicts a re-equilibration of the rise in surface tension $\Delta\gamma$ upon application of the drain over a time course of approx. 300 seconds, in agreement with the experimental observations in **Figure 6a**. The value of α corresponds to the surface/volume ratio for a solution with a height of 0.5 cm. The value of A_s corresponds to the content of 1 μL $C_{12}E_4$ (density 946 g L^{-1}; molecular weight 362.5 g mol^{-1}), spread over the area of the petri dish ($a = 9.6$ cm^2).

To simulate the surface tension kinetics in **Figure 2e-f**, $A_s = 0$ as there is no amphiphile source droplet present in the simulation. We assume that the amphiphile concentration in the aqueous solution A_m is constant and, due to a large excess of $C_{12}E_4$ (0.52 mM; $A_m = 5.2\cdot10^{-7}$ mol cm^{-3}), does not change upon depletion from the air-water interface by a drain droplet that is deposited at $t = 0$ s. We also assumed that the amphiphiles in the $C_{12}E_4$ micelles take part (*i.e.* enhance) the kinetics of the surface tension equilibration, as they re-occupy vacant sites when a drain is applied that depletes amphiphiles from the air-water interface. To take into account that the maximum occupation of amphiphiles at the air-water interface does not exceed Γ_{CMC}, we implied $\Gamma(t = 0) = \frac{A_m\cdot\Gamma_{CMC}}{K_3 + A_m}$ and $\theta(t = 0) = \Gamma_{CMC} - \Gamma(t = 0)$ as an approximation.

Simulate flow patterns: An important assumption in the simulation of the flow profile based on the rate of amphiphile depletion from the air-water interface towards the underlying aqueous phase (Φ_{water}), is that this depletion predominantly occurs via the Marangoni flow. At the boundary of the flow-zone, a downward flow emerges that transfers amphiphile from the air-water interface towards the underlying bulk solution. We considered that the change in Γ along the flow pattern from the source droplet towards the boundary of the flow zone is very small: In the PIV analysis, we observed typical flow rates up to 20 μm s^{-1}. Via the relationship $\eta(\mathrm{d}v/\mathrm{d}z) = (\mathrm{d}\gamma/\mathrm{d}x)$, with viscosity $\eta = 5$ mPa.s for the NaAlg/water solution[5] and velocity $v = 0$ at z = –1 mm from the air-water interface (as observed in our measurements, **Figure S3d**), we estimate only a very minor surface tension gradient $\mathrm{d}\gamma/\mathrm{d}x = 1\cdot10^{-3}$ mN m^{-1} cm^{-1} – *i.e.* a surface tension difference $\Delta\gamma = 1.75\cdot10^{-3}$ mN m^{-1} between the location of the source and the edge of the petri dish with a radius of 17.5 mm. Via the Frumkin isotherm (eq. 8), this value of $\Delta\gamma$ corresponds to a difference in surfactant concentration at the air-water interface $\Delta\Gamma = 2.7\cdot10^{-15}$ mol cm^{-2}, which is multiple orders of magnitude lower than the surfactant concentration $\Gamma = 4.458\cdot10^{-10}$ mol cm^{-2}. The

difference in surfactant density at the air-water interface $\Delta\Gamma$ times the area of the ring that disappears via convection at the boundary of the interface is then the amount of surfactant per second that has been removed from the interface upon diffusion to the bulk, while the flow progresses towards the boundary of the system. As a result, the depletion rate via direct diffusion towards the underlying aqueous phase is approximately $\Delta\Gamma \cdot 2\pi R v_{\text{source}}(17.5\text{ mm}) = 3\cdot10^{-18}\text{ mol s}^{-1}$, with $R = 17.5$ mm and $v_{\text{source}}(17.5\text{ mm}) = 1\cdot10^{-3}\text{ mm s}^{-1}$ (the velocity at the boundary of the flow zone, as simulated in Figure 2). This depletion rate is, again, orders of magnitude lower than the depletion rate predicted in **Figure 2d** ($4.2\cdot10^{-13}$ mol s^{-1}). As a result, only a very small portion of Γ is depleted as the flow progresses towards the edge of the flow zone to maintain this surface tension gradient, implying that the rest of the amphiphile is depleted to the aqueous phase via the counter-flow.

Next, we calculate $v_{\text{source}}(R)$ at the edge of the flow zone with radius R via $\Phi_{\text{water}} \cdot a = \Gamma \cdot a_{\text{ring}}$, with $a_{\text{ring}} = 2\pi R \cdot v_{\text{source}}(R) \cdot (1\text{ s})$ and a the total area of the air-water interface. Here, we assume that $v_{\text{source}}(R) \cdot (1\text{ s}) << R$. This yields:

(13) $$v_{\text{source}}(R) = \frac{\Phi_{water} \cdot a}{2\pi\Gamma R}.$$

And via the relationship $v \sim r^{n}$, one can find:

(14) $$v_{\text{source}}(r) = v_{\text{source}}(R)\left(\frac{r}{R}\right)^{n}.$$

In analogy, we define for the flow towards the drain v_{drain}:

(15) $$v_{\text{drain}}(R_{\text{drain}}) = -\frac{\Phi_{\text{drain}} \cdot a}{2\pi\Gamma R_{\text{drain}}};$$

(16) $$v_{\text{drain}}(r_{\text{drain}}) = v_{\text{drain}}(R_{\text{drain}})\left(\frac{r_{\text{drain}}}{R_{\text{drain}}}\right)^{n}.$$

Here, R_{drain} equals the radius of the drain. Importantly, we included a (–) sign in the expression for v_{drain} (eq. 15), since the flow velocity is oriented towards the drain, whereas the v_{source} is directed away from the source.

For every (x,y) coordinate, we calculate r via $r = \sqrt{(x - x_{\text{source}})^2 + (y - y_{\text{source}})^2}$, and r_{drain} via $r_{\text{drain}} = \sqrt{(x - x_{\text{drain}})^2 + (y - y_{\text{drain}})^2}$, where x_{source} and y_{source} represent the x- and y-positions of the source, and x_{drain} and y_{drain} the x- and y-positions of the drain, respectively.

Next, the x- and y-components of v_{source} at (x,y) are found via:

(17) $$v_{\text{source,x}}(x,y) = \frac{v_{\text{source}}(r) \cdot (x - x_{\text{source}})}{\sqrt{(x - x_{\text{source}})^2 + (y - y_{\text{source}})^2}};$$

(18) $$v_{\text{source,y}}(x,y) = \frac{v_{\text{source}}(r) \cdot (y - y_{\text{source}})}{\sqrt{(x - x_{\text{source}})^2 + (y - y_{\text{source}})^2}};$$

and the x- and y-components of v_{drain} at (x,y) via:

$$(19) \qquad v_{\mathrm{drain,x}}(x,y) = \frac{v_{\mathrm{drain}}(r_{\mathrm{drain}}) \cdot (x - x_{\mathrm{drain}})}{\sqrt{(x - x_{\mathrm{drain}})^2 + (y - y_{\mathrm{drain}})^2}} \;;$$

$$(20) \qquad v_{\mathrm{drain,y}}(x,y) = \frac{v_{\mathrm{drain}}(r_{\mathrm{drain}}) \cdot (y - y_{\mathrm{drain}})}{\sqrt{(x - x_{\mathrm{drain}})^2 + (y - y_{\mathrm{drain}})^2}} \,.$$

Then, the x-component of the velocity $v_{\mathrm{x}}(x,y)$ equals $v_{\mathrm{source,x}}(x,y) + v_{\mathrm{drain,x}}(x,y)$, and the y-component $v_{\mathrm{y}}(x,y)$ equals $v_{\mathrm{source,y}}(x,y) + v_{\mathrm{drain,y}}(x,y)$, such that the total flow velocity $v(x,y) = \sqrt{v_x(x,y)^2 + v_y(x,y)^2}$.

The simulations shown in **Figure 2** where performed in Matlab (R2017a). First, based on the kinetic rate constants, Φ_{water} and Φ_{drain} were simulated via the kinetic model discussed above, using the *ode15s solver*. Next, we computed the flow velocity and direction at every position (x, y) in a 200x200 matrix that represents a square of 35 by 35 mm^2, with a source positioned at $(x_{\mathrm{source}}, y_{\mathrm{source}})$ = (17.5 mm, 17.5 mm) and a drain at $(x_{\mathrm{drain}}, y_{\mathrm{drain}})$ = (26.35 mm, 17.5 mm). The heat maps represent the flow velocity within a radius of 17.5 mm around the source; the red curves indicate the streamlines (plotted via the *streamline* function in Matlab) from the source towards the drain.

The simulations are performed with R_{drain} = 0.5 mm and n = - 1. The simulations shown in **Figure 2** are performed with the radius of the flow zone equal to R = 17.5 mm. In **Figure S2**, we performed the simulations with the radius of the flow zone equal to 8 mm. Here, the term R in eq. 13 and 14 is set to 8 mm, while the radius of the system (17.5 mm) and the positions of the source and drain are kept the same.

3. Supporting Figures

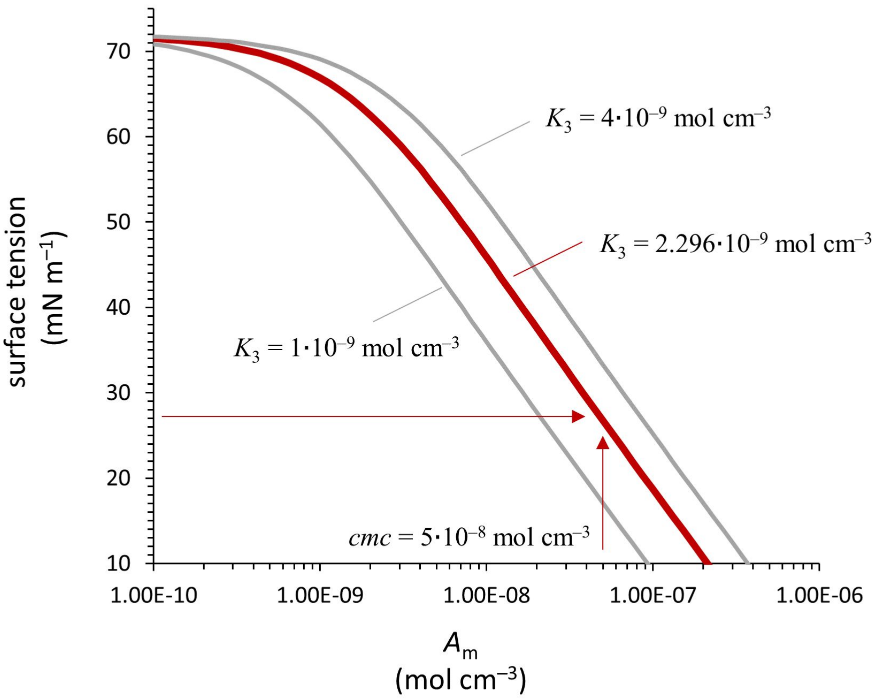

Figure S1. Surface tension *vs.* concentration of amphiphile A_m, computed with different values of K_3. We computed Γ based on A_m via equation 11, and subsequently the surface tension γ based on Γ via equation 8. With larger values of K_3, the surface tension decreases faster with A_m. With $K_3 = 2.296 \cdot 10^{-9}$ mol cm^{-3}, eq. 11 and eq. 8 predict a surface tension of 27 mN m^{-1} at $A_m = 5 \cdot 10^{-8}$ mol cm^{-3}, as was reported in literature.[6] Importantly, this graph is intended to assess how varying K_3 affects the predicted surface tension at the cmc = $5 \cdot 10^{-8}$ mol cm^{-3}. The levelling off of the surface tension ($\gamma = \gamma_{cmc}$) beyond the cmc is not included in eq. 8 and eq. 11.

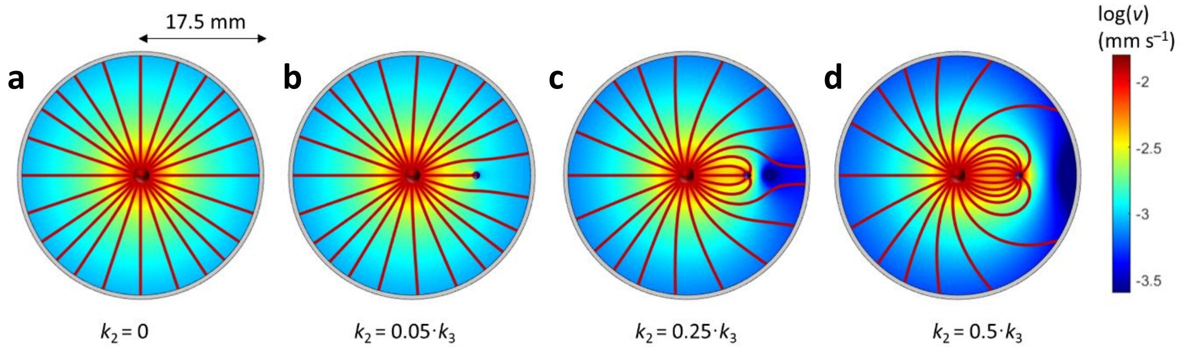

Figure S2. Flow velocity profiles simulated with a smaller flow zone (*R* = 8 mm). In this Supporting Figure, the simulations are performed with the radius of the flow zone $R = 8$ mm. This assumes that the ring where the desorption of the surfactants to the underlying aqueous phase occurs (in the absence of the drain) has a radius of 8 mm (rather than being positioned at the boundary of the petri dish). However, the overall size of the system is still the same, *i.e.* $r = 17.5$ mm. The simulations are performed with similar parameters as the simulations shown in Figures 2g-j.

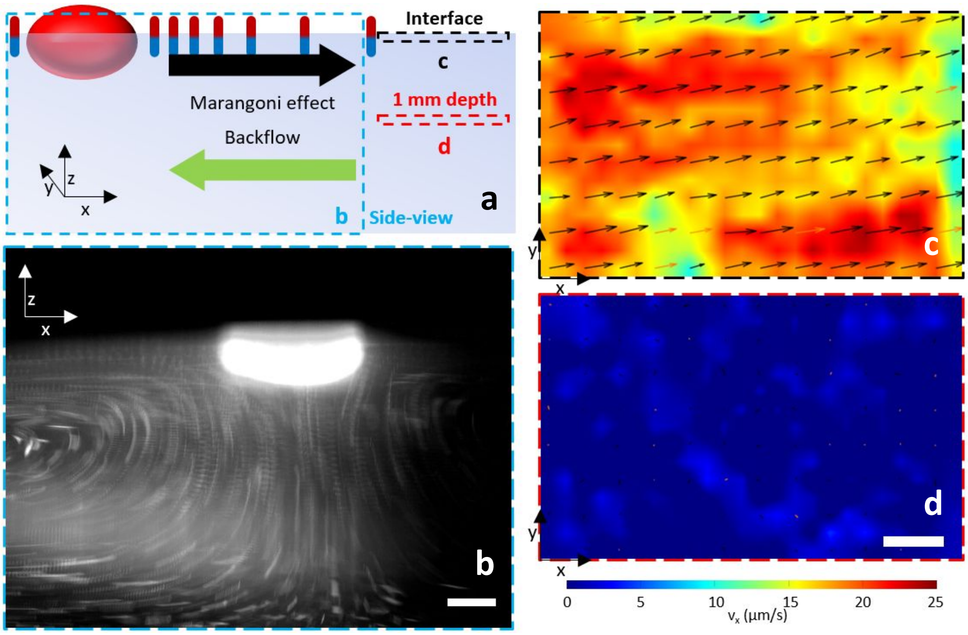

Figure S3. Analysis of the flow in the aqueous solution surrounding a $C_{12}E_4$ source droplet at various depths. a) Schematic representation of the Marangoni flow induced by the source at the air-water interface and the resulting backflow at depth > 1 mm below the interface. **b)** Fluorescence microscopy time-lapse image (side view) compiled over 15 seconds. The flow profile is visualized by fluorescent polystyrene beads, which reveal that the flow is directed away from the source near the interface and towards the source near the bottom of the petri dish. **c-d)** PIV analysis of the flow velocity in the x-direction at the air-water interface (**c**) and at 1 mm depth (**d**), revealing average flow velocities of 18 µm/s and 80 nm/s, respectively. The scale bar in (**b**) represents 500 µm, the scale bar corresponding to (**c**) and (**d**) represents 100 µm.

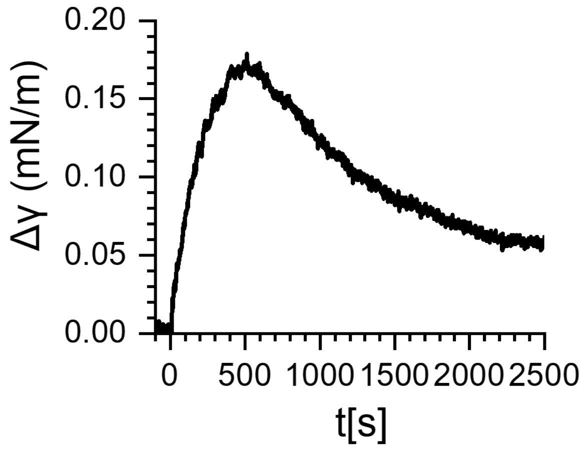

Figure S4. Surface tension evolution for a 10% NaOleate/OA drain on a 0.52 mM $C_{12}E_4$ / 17 mM NaCl solution. After deposition of the drain at t = 0 s, a lasting increase in surface tension is observed, indicating a continuous uptake of $C_{12}E_4$ molecules from the air-water interface by the droplet.

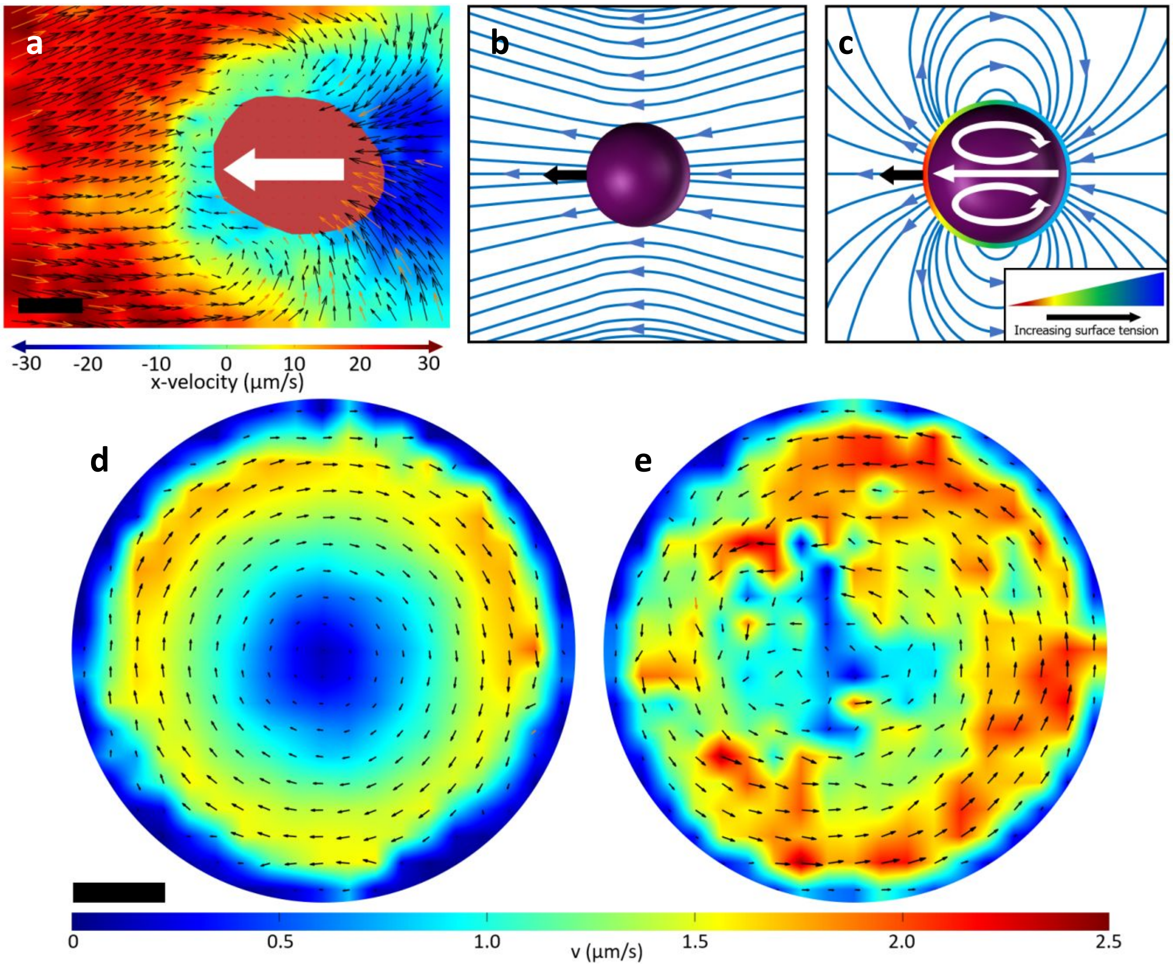

Figure S5. PIV study of the flow profile around and inside drain droplets. a) PIV analysis of the flow surrounding a 10 wt% NaOleate/OA drain droplet as it moves towards the source. The liquid near the drain moves in parallel with the drain, matching (**b**) the schematic flow profile that would be expected for a sphere that is moved by an external force.[7] **c**) Schematic internal (white arrows) and external (blue arrows) flow profiles corresponding to a droplet undergoing Marangoni-driven self-propulsion.[7,8] At the sides of the droplet, the medium moves towards the backside of the droplet, opposite to the direction in which the droplet moves. **d-e)** Average PIV analysis of the flow profile inside a 10% NaOleate/OA drain (**d**) and a 10% $C_{12}E_4$/OA drain (**e**). The drains move continuously towards the source over the course of this PIV analysis (11 seconds). While the droplet as a whole rotates slightly, no consistent internal flow patterns are observed. The scale bars in (**a**) and (**d**) represent 500 μm and 200 μm, respectively.

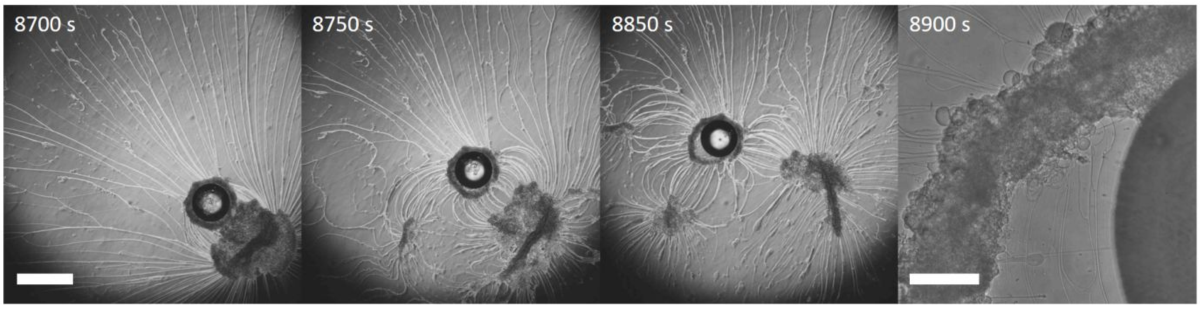

Figure S6. Filaments cluster into a ring-like structure around the drain. Optical microscopy images of 20% $C_{12}E_4$/OA drain droplets. After a $C_{12}E_4$ source droplet is made to burst by rapidly lifting the covering petri dish (see Methods for details), the fragments of the source pull on the filament cluster from different directions, causing it to partially release from the drain. The drain was deposited at $t = 0$ s. The left scale bar represents 2 mm; the right scale bar represents 200 μm.

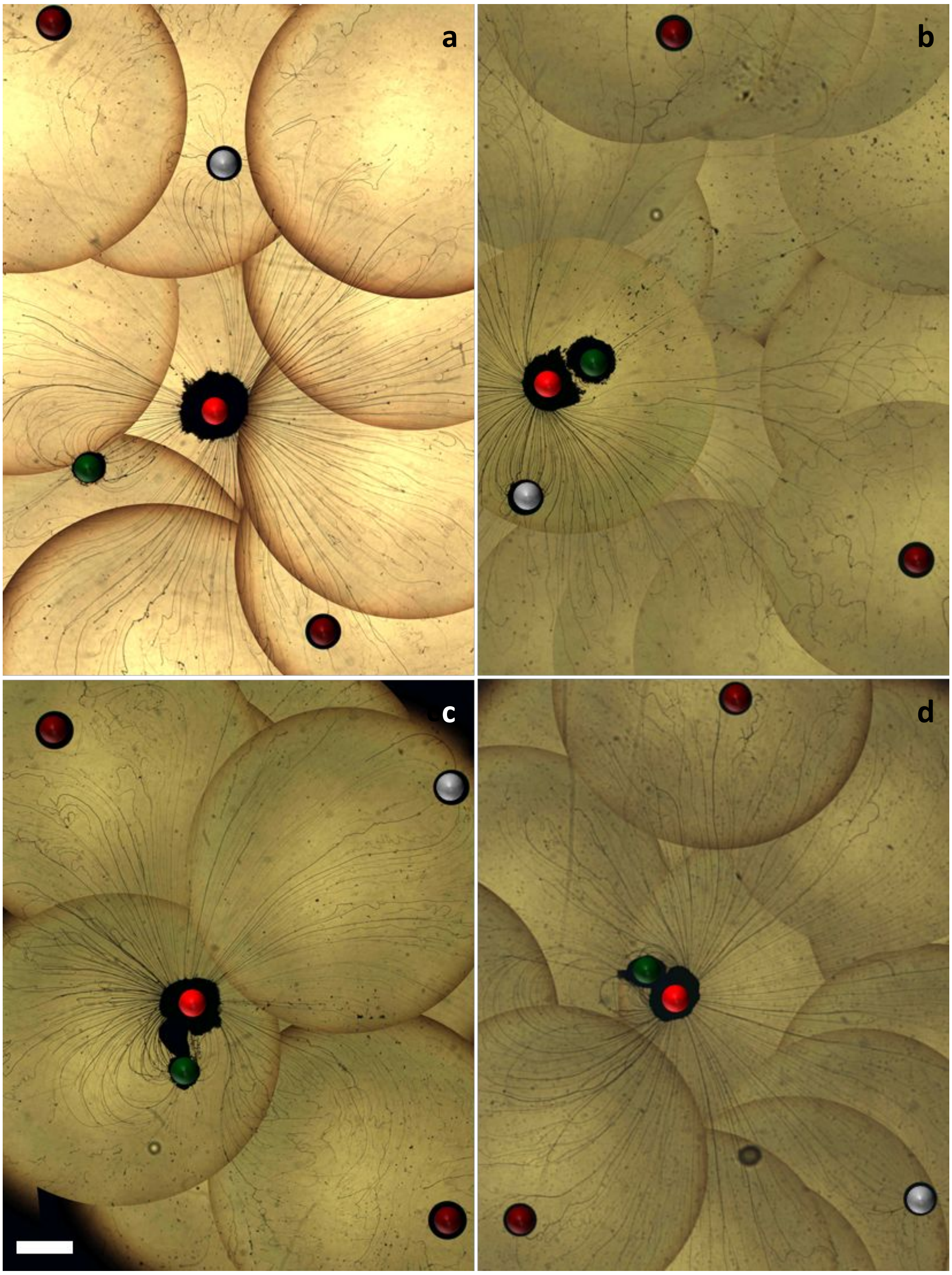

Figure S7. Additional experiments with selective attraction of drain droplets towards the source by filaments. a-d) Composed optical microscopy image of one source droplet (light red) with four OA-based drains including 30% $C_{12}E_4$ (dark red, 2x), 20% $C_{12}E_4$ (grey) and 10% $C_{12}E_4$ (green). The images are acquired 19, 23, 15 and 27 min. after droplet deposition for a, b, c and d, respectively. The scale bar represents 2 mm.

4. Description of Supporting Movies

For all movies displaying fluorescence microscopy, contrast was enhanced using Fiji such that 0.3% of all pixels in the image are saturated.

File Name: Movie S1

Description: Top-view fluorescence microscopy movie corresponding to Figure 3a. Fluorescent particles suspended in solution visualize the flow of the medium around a 1.0 µL $C_{12}E_4$ source droplet.

File Name: Movie S2-3

Description: Top-view fluorescence microscopy movie corresponding to Figure 3d. 1.0 µL of 10% NaOleate/OA drain solution has been deposited at $t = 0$ s onto an air/water interface on which a 1.0 µL $C_{12}E_4$ source droplet was already present. In Movie S2, the stationary drain induces a roughly symmetrical flow, as indicated by the movement of fluorescent particles. In Movie S3, filaments have attached to the drain, which now moves towards the source droplet until a distance of approximately 500 µm is reached.

File Name: Movie S4

Description: Bottom-view optical microscopy movie corresponding to Figure 4c-d. 1.0 µL of 10% NaOleate/OA (left) and 1.0 µL of 10% $C_{12}E_4$/OA (right) were deposited onto an air/water interface on which a 1.0 µL $C_{12}E_4$ source droplet containing 5.5 mg/mL Oil Red O was already present. Left: the dye is not absorbed, even upon collision with the source, as filaments cluster around the drain. Right: the drain droplet moves rapidly towards the source, absorbing the red dye along with the filaments in which it is contained.

File Name: Movie S5

Description: Bottom-view optical microscopy movie corresponding to Figure 4e. First, a 1.0 µL 20% $C_{12}E_4$/OA drain droplet is deposited, followed by deposition of a 1.0 µL $C_{12}E_4$ source. Zooming in on the drain, we observe how filaments cluster and wrap around the drain droplet as it approaches the source.

File Name: Movie S6

Description: Bottom-view optical microscopy movie corresponding to Figure 5a. First, a 1.0 µL $C_{12}E_4$ source droplet is deposited, followed by deposition of a MolSieve drain at t = 0 s. The drain initially moves away from the source, but eventually filaments attach to keep it in place and even attract the MolSieve slightly towards the source.

File Name: Movie S7

Description: Top-view fluorescence microscopy movie corresponding to Figure 6e. First, a 1.0 µL $C_{12}E_4$ source droplet is deposited, followed by deposition of 1.0 µL $C_{12}E_4$/OA drain solution at $t = 0$ s (left: 10% $C_{12}E_4$/OA; middle: 20% $C_{12}E_4$/OA; right: 30% $C_{12}E_4$/OA). The drain moves towards the source while fluorescent particles suspended in solution visualize the flow of the medium. The Marangoni flow caused by the drain is visualized through the movement of fluorescent particles.

File Name: Movie S8

Description: Bottom-view optical microscopy movie corresponding to Figure 6f. First, a 1.0 µL $C_{12}E_4$ source droplet is deposited, followed by deposition of 1.0 µL $C_{12}E_4$/OA drain solution at $t = 0$ s (left: 10% $C_{12}E_4$/OA; middle: 20% $C_{12}E_4$/OA; right: 30% $C_{12}E_4$/OA). 10% $C_{12}E_4$/OA and 20% $C_{12}E_4$/OA drains generate a Marangoni flow that is strong enough to attract filaments which pull the drain towards

the source, while filaments are repelled from the 30% $C_{12}E_4$/OA drain. At t = 1826 s, the petri dish covering the setup is removed in the left panel, resulting in destabilization of the source droplet.

File Name: Movie S9

Description: Side-view fluorescence microscopy movie corresponding to Figure S3b. Fluorescent particles suspended in solution visualize the flow of the medium around a 1.0 µL $C_{12}E_4$ source droplet.

File Name: Movie S10-11

Description: Top-view fluorescence microscopy movie corresponding to Figures S3c-d. Fluorescent particles suspended in solution visualize the flow of the medium at 5 mm distance from a 1.0 µL $C_{12}E_4$ source droplet at the air-water interface (Movie S10) and 1 mm below the air-water interface (Movie S11).

File Name: Movie S12-13

Description: Fluorescence microscopy movie corresponding to Figures S5d-e. 1.0 µL of 10% NaOleate/OA (Movie S12) and 10% $C_{12}E_4$/OA (Movie S13) were deposited, onto an air/water interface on which a 1.0 µL $C_{12}E_4$ source droplet was already present. Fluorescent particles visualize the movement of the liquid inside the drain droplets. Notably, no consistent flow is observed inside either droplet, although a flow pattern emerges momentarily inside the 10% $C_{12}E_4$/OA drain at t = 134.5 s.

File Name: Movie S14

Description: Optical microscopy movie corresponding to Figure S6. A 1.0 µL $C_{12}E_4$ source and 1.0 µL 20% $C_{12}E_4$/OA drain droplet were deposited. Upon lifting the covering petri dish at t = 8698s, the source droplet bursts into multiple fragments and the filament cluster partially detaches from the drain.

5. References

(1) Thielicke, W.; Stamhuis, E. J. PIVlab – Towards User-Friendly, Affordable and Accurate Digital Particle Image Velocimetry in MATLAB. *J. Open Res. Softw.* **2014**, *2*, e30.

(2) Thielicke, W.; Sonntag, R. Particle Image Velocimetry for MATLAB: Accuracy and Enhanced Algorithms in PIVlab. *J. Open Res. Softw.* **2021**, *9* (May), 1–14.

(3) Tinevez, J. Y.; Perry, N.; Schindelin, J.; Hoopes, G. M.; Reynolds, G. D.; Laplantine, E.; Bednarek, S. Y.; Shorte, S. L.; Eliceiri, K. W. TrackMate: An Open and Extensible Platform for Single-Particle Tracking. *Methods* **2017**, *115*, 80–90.

(4) Schindelin, J.; Arganda-Carreras, I.; Frise, E.; Kaynig, V.; Longair, M.; Pietzsch, T.; Preibisch, S.; Rueden, C.; Saalfeld, S.; Schmid, B.; Tinevez, J. Y.; White, D. J.; Hartenstein, V.; Eliceiri, K.; Tomancak, P.; Cardona, A. Fiji: An Open-Source Platform for Biological-Image Analysis. *Nat. Methods* **2012**, *9* (7), 676–682.

(5) van der Weijden, A.; Winkens, M.; Schoenmakers, S. M. C.; Huck, W. T. S.; Korevaar, P. A. Autonomous Mesoscale Positioning Emerging from Myelin Filament Self-Organization and Marangoni Flows. *Nat. Commun.* **2020**, *11*, 4800.

(6) Hsu, C. T.; Shao, M. J.; Lin, S. Y. Adsorption Kinetics of C12E4 at the Air-Water Interface: Adsorption onto a Fresh Interface. *Langmuir* **2000**, *16* (7), 3187–3194.

(7) Zöttl, A.; Stark, H. Emergent Behavior in Active Colloids. *J. Phys. Condens. Matter* **2016**, *28* (25), 253001.

(8) Herminghaus, S.; Maass, C. C.; Krüger, C.; Thutupalli, S.; Goehring, L.; Bahr, C. Interfacial Mechanisms in Active Emulsions. *Soft Matter* **2014**, *10* (36), 7008–7022.
